# Multi‐miRNAs‐Mediated Hepatic Lepr Axis Suppression: A Pparg–Dicer1 Pathway‐Driven Mechanism in Spermatogenesis for the Intergenerational Transmission of Paternal Metabolic Syndrome

**DOI:** 10.1002/advs.202410831

**Published:** 2025-01-10

**Authors:** Yi Lin, Xiuye Ni, Lin Zhu, Yilong Lin, Cai Peng, Zhao Lei, Yihui Wang, Huan Wang, Xiang You, Juan Li, Heqing Shen, Jie Wei

**Affiliations:** ^1^ State Key Laboratory of Vaccines for Infectious Diseases， Xiang An Biomedicine Laboratory， National Innovation Platform for Industry‐Education Integration in Vaccine Research School of Public Health Xiamen University Xiamen 361102 China; ^2^ Department of Basic Medical Sciences School of Medicine Xiamen University Xiamen 361102 China

**Keywords:** bisphenol A, intergenerational transmission, leptin resistance, metabolic syndrome, microRNAs

## Abstract

Bisphenol A (BPA) is an “environmental obesogen” and this study aims to investigate the intergenerational impacts of BPA‐induced metabolic syndrome (MetS), specifically focusing on unraveling mechanisms. Exposure to BPA induces metabolic disorders in the paternal mice, which are then transmitted to offspring, leading to late‐onset MetS. Mechanistically, BPA upregulates Srebf1, which in turn promotes the Pparg‐dependent transcription of *Dicer1* in spermatocytes, increasing the levels of multiple sperm microRNAs (miRNAs). Several of these miRNAs are highly expressed in a synchronized manner in liver of the offspring. miR149‐5p, miR150‐5p, and miR700‐5p target a specific region in the Lepr 3′UTR, termed “SMITE” (“Several MiRNAs Targeting Elements”), to negatively regulate Lepr. These inherited anti‐Lepr miRNAs, also referred to inherited anti‐Lepr miRNAs (IAL‐miRs), modulate hepatic steatosis, and insulin signaling through the Lepr regulatory Igfbp2, Egfr, and Ampk. Furthermore, IAL‐miRs inhibit Ccnd1 not only via binding to “SMITE” but also via Lepr–Igfbp2 axis, which contribute to hepatocyte senescence. These pathological processes interact in a self‐reinforcing cycle, worsening MetS in the paternal BPA‐exposed offspring. The findings reveal mechanism wherein lipid metabolism reprogramming in spermatocytes‐induced perturbations of sperm miRNAs, triggered by BPA, leads to intergenerational inheritance of paternal MetS through suppression of the hepatic Lepr axis in the offspring.

## Graphical abstract

Metabolic syndrome (MetS) arises from the complex interplay between genetic and environmental factors. Bisphenol A exposure triggers perturbations of sperm microRNAs (miRNAs) in the paternal parent by reprogramming lipid metabolism in spermatocytes. Inherited miRNAs suppress hepatic Lepr via binding to “SMITE” in the offspring, leading to the intergenerational transmission of paternal MetS.

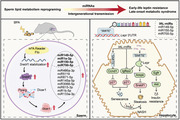

## 1 Introduction

Metabolic syndrome (MetS) is a widespread health problem characterized by a combination of factors, including abdominal obesity, hyperlipidemia, insulin resistance (IR), and hypertension. The prevalence of MetS has escalated to epidemic proportions within the last four decades.^[^ ^1^ ^]^ In conjunction with lifestyle factors such as an unhealthy diet, sedentary habits, smoking, or alcohol intake, exposure to exogenous environmental contaminants initiates or substantially contributes to the onset of MetS. Bisphenol A (BPA) is a widely studied environmental metabolism‐disrupting chemical (MDC). We and others have identified links between BPA exposure and glucose and lipid metabolism disorders both in vitro and in vivo.^[^ ^2^ ^]^ BPA is best known for its estrogenicity, having been demonstrated to bind to classical estrogen receptors (ERs), namely, ERα and ERβ, androgen receptor, as well as to nonclassical plasma membrane‐bound ERs such as the G‐protein‐coupled receptor 30 (GPR30). Moreover, BPA interacted with various nuclear receptors, including the thyroid receptor, glucocorticoid receptor, pregnane X receptor, aryl hydrocarbon receptor, and peroxisome proliferator‐activated receptor gamma (Pparg).^[^ ^3^ ^]^ These binding interactions are associated with enhanced adipogenesis, suppression of insulin secretion in pancreatic islets, and impaired insulin signaling in peripheral tissues, thereby contributing to the pathogenesis of obesity and IR. In addition, aberrant redox signaling in metabolic organs such as the liver, adipose tissue, skeletal muscle, and pancreas also underlies the pathophysiology of MetS. For example, we previously established that mitochondrial defects and mitochondria‐dependent apoptotic signals in islet β‐cells were crucial contributors to the pathogenesis of BPA‐induced glucometabolic disorders.^[^ ^2b^ ^]^ The potential mechanism by which BPA induces MetS also involves the chronic inflammation.^[^ ^4^ ^]^ BPA increased de novo ceramide synthesis, promoting the secretion of proinflammatory cytokines through the JNK/NF‐kB signaling pathway, thereby exacerbating metabolic derangement during obesity.^[^ ^5^ ^]^ Furthermore, exposure to BPA can induce epigenetic modifications, including DNA methylation, histone acetylation, and changes in noncoding RNAs, which are associated with the regulation of genes associated with MetS.^[^ ^6^ ^]^ These epigenetic signatures contribute to the transgenerational inheritance of BPA‐induced metabolic disorders.

Indeed, the developmental origins of health and disease (DOHaD) hypothesis suggests that exposure to MDCs during prenatal or early childhood is associated with an elevated risk of developing metabolic diseases later in life, thereby perpetuating the cycle of disease susceptibility across generations. MDCs can effectively “reprogram” germ line cells. Our previous studies and the majority of current studies have focused primarily on maternal exposure. We showed that perinatal exposure to BPA predisposed the adult offspring to develop obesity, IR, and hepatic steatosis later in life.^[^ ^7^ ^]^ However, when exposure occurs during pregnancy and lactation, factors such as the transplacental transport of BPA, the presence of BPA in breast milk, the fetus's adaptation to the intrauterine environment, and maternal caregiving behaviors will also influence the reprogramming of the offspring's health, extending beyond the detrimental effects on the germline. To better understand the mechanisms underlying the intergenerational transmission of phenotypes, we need to identify how and when epigenetic perturbations in germ cells influence offspring.^[^ ^8^ ^]^ In contrast to females, paternal contribution is limited to the provision of sperm, with males not typically engaging in direct physiological or behavioral interactions with the fetus. Multiple animal models have demonstrated that the paternal diet, psychological stress, and environmental exposures lead to metabolic disorders in the offspring.^[^ ^9^ ^]^ Human epidemiological studies also provided evidence that offspring of paternal areca chewers were more prone to MetS than those fathers abstained from chewing, even if the offspring themselves did not chew.^[^ ^10^ ^]^ For BPA, only one study reported that BPA‐exposed male mice sired female offspring exhibiting impaired glucose tolerance and highlighted that diabetes in BPA‐exposed fathers may be a prerequisite for phenotypic transmission.^[^ ^11^ ^]^ Whether or how paternal BPA exposure affects the development of MetS in offspring requires further exploration.

Sperm‐derived epigenetic changes, including DNA methylation patterns, retained histone profiles, and small noncoding RNA transcriptomes, contribute to the intergenerational transmission of disease phenotypes, irrespective of DNA damage and de novo genetic mutations. We have previously demonstrated that microRNAs (miRNAs) have the potential to serve as biomarkers and regulators of metabolic diseases and disorders triggered by BPA. In the liver, BPA increased de novo lipogenesis by inhibiting miR192, consequently leading to the development of nonalcoholic fatty liver disease (NAFLD).^[^ ^2a^ ^]^ By targeting Pdx‐1, miR338 controlled the BPA‐induced pancreatic insulin secretory of epigenetic information transmission, some miRNAs have been reported to exhibit consistent changes in the liver of father‐offspring pairs of animals. Moreover, these changes were accompanied by the transmission of paternal phenotypes with abnormal cholesterol and lipid metabolism in the offspring.^[^ ^12^ ^]^ In a chronic mild stress‐induced depression‐like mouse model, different miRNAs expression profiles were also observed in the sperm of F0 mice, and neutralizing of these abnormal miRNAs in zygotes rescued the acquired depression‐like phenotypes in F1 offspring.^[^ ^13^ ^]^ Similarly, paternal stress epigenetically downregulated miR466b‐3p, leading to increased Pck1 expression and hepatic gluconeogenesis, ultimately leading to hyperglycemia in F1 mice.^[^ ^9c^ ^]^ Therefore, sperm can transmit miRNAs‐based epigenetic signals from the father to the offspring while also displaying sensitivity to environmental stimuli.^[^ ^14^ ^]^

In this study, we investigated the sperm miRNAs profiles of BPA‐exposed mice, with a specific focus on exploring the underlying mechanisms responsible for sperm epigenomic alterations. Additionally, we sought to determine the inheritance patterns of these alterations and their potential influence on the susceptibility of offspring to MetS. The findings should help to offer novel insights into the mechanisms underlying the intergenerational transmission of MetS resulting from paternal exposure to BPA, which have not been previously investigated.

## 2 Results

### 2.1 The BPA‐Exposed F0 Male Mice Exhibited a MetS‐Like Phenotype and Experienced Alterations in Sperm miRNA Profile

**Figure 1:**
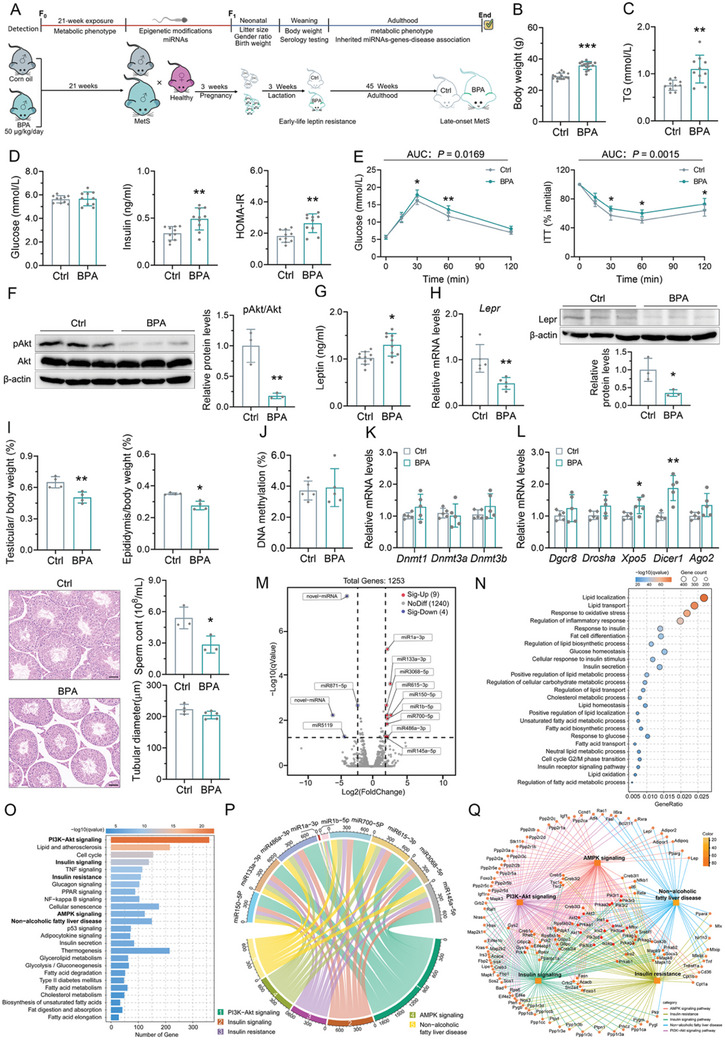
Altered sperm miRNAs profile in BPA‐exposed mice with MetS‐like symptoms. A) Schematic illustration of the experimental design. B) The body weight after 21‐week BPA exposure. C) Serum triglycerides (TG). D) Levels of fasting glucose and insulin. HOMA‐IR was calculated by (fasting glucose [mmol L−1] × fasting insulin [mIU mL^−1^])/22.5. E) The dynamics of the blood glucose curve during IPGTT and ITT. The area under the curve (AUC) values was calculated and showed in Figure S1D of the Supporting Information. F) The expression levels of pAKT and AKT and the quantitation of pAKT/AKT ratios in the liver. G) Serum leptin concentration. H) The mRNA and protein expression of Lepr in the liver. Data were normalized to β‐actin. I) The organ index for testis and cauda epididymis, sperm count, and representative morphology of testis. The organ index was the ratio of weight of testis or epididymis over body weight, and the absolute weight were showed in Figure S1E of the Supporting Information. The tubular diameter was quantified to assess changes in testis structure. J) The global DNA methylation in sperm. K) The mRNA expression of DNA methyltransferases. The β‐actin was used as a loading control. L) The mRNA expression of genes involved in the miRNAs biogenesis. The β‐actin was used as a loading control. M–Q) The sperm small noncoding RNA (sncRNAs)‐sequencing data. The principal component analysis (PCA) used to examine the variance in sperm miRNAs distribution between control and BPA‐exposed F0 male mice were showed in Figure S2E of the Supporting Information. (M) The volcano plot illustrated the biological and statistical significance of differentially expressed miRNAs between control and BPA‐exposed mice. A fourfold threshold was used to screen the differentially expressed miRNAs. The cluster heat map of the differential miRNAs was showed in Figure S2F of the Supporting Information. (N) The enriched GO‐BP classification histogram belonged mainly to glucose and lipid metabolism pathways. The top 25 enriched GO biological process (GO‐BP), cellular component (GO‐CC), and molecular function (GO‐MF) terms were showed in Figure S2G–I of the Supporting Information. Color scale indicated ‐log (*q*‐value) of enrichment. Count, differential gene count in the indicated pathway; GeneRatio, differential gene count in the indicated pathway versus total differential gene count. (O) Top 25 significantly enriched KEGG pathways of differential miRNAs target genes. Levels of significance of enrichment in each pathway represented by ‐log (*q*‐value). The pathways related to glucose and lipid metabolism were considered as hub pathways and bolded. (P) Chord diagram summarizing the regulation of differentially expressed miRNAs and their corresponding KEGG pathways that are associated with glucose and lipid metabolism. The arcs on the top represented the differentially expressed miRNAs, and the arcs on the bottom showed the pathways. The width of the links between them indicates the number of the predicted target genes of differentially expressed miRNAs and the color of the link matched the color of the pathways. (Q) The interaction network of predicted target genes of differentially expressed miRNAs in hub KEGG pathways. The square represented the hub KEGG pathways and the circle represented the predicted target genes of differentially expressed miRNAs. All data in this figure were from F0 male mice. *n* = 3–10 mice per group as indicated. Data were presented as the mean ± SD. ^*^ *p* < 0.05, ^**^ *p* < 0.01, ^***^ *p* < 0.001, versus corresponding controls.

As depicted in **Figure** **1**A, male C57B/L6 mice (F0) were fed either a control diet or an identical diet supplemented with 0.5 µg g^−1^ of BPA for 21 weeks. BPA exposure increased body weight (Figure 1B; Figure S1A, Supporting Information), concurrently leading to elevated serum triglycerides (TG) levels (Figure 1C). However, the serum TC (total cholesterol), AST, and ALT levels did not differ between the groups (Figure S1B,C, Supporting Information). Despite not detecting changes in fasting blood glucose, both the serum insulin and the indices of IR, such as homeostasis model assessment for IR (HOMA‐IR), were increased in BPA‐exposed F0 male mice (Figure 1D). The OGTT and ITT also showed that exposure to BPA caused whole‐body glucose intolerance and impaired insulin sensitivity in F0 male mice (Figure 1E; Figure S1D, Supporting Information). Furthermore, BPA exposure inhibited insulin signaling in the liver of F0 male mice as evidenced by decreased phosphorylated Akt expression (Figure 1F). Then, we further measured serum leptin which is typically correlated with metabolic reprogramming and demonstrated that fasting serum leptin was elevated (Figure 1G) while the hepatic expression of Lepr was reduced (Figure 1H) in the BPA‐exposed F0 male mice.

Germ cells might mediate the intergenerational transmission of these acquired paternal characteristics. Exposure to BPA resulted in a lower relative weight of the testis and cauda epididymis, as well as reduced sperm count in epididymis tissues (Figure 1I; Figure S1E, Supporting Information). Hematoxylin and eosin (H&E) staining of the testes showed that the gaps between seminiferous tubules were enlarged in the BPA‐exposed mice (Figure 1I). Next, we determined there were epigenetic alterations present in the sperm after exposure to BPA. Figure 1J,K indicated that neither the level of global DNA methylation nor the expression of the DNA methyltransferases *Dnmt1*, *Dnmt3a*, and *Dnmt3b* was altered by BPA. However, BPA upregulated *Xpo5* and *Dicer1*, which are the key components of miRNAs processing and biogenesis pathway (Figure 1L). miRNA expression profiles derived from sperm were thus analyzed using small RNA sequencing. PCA demonstrated that BPA induced significant perturbations in sperm miRNAs (Figure S2E, Supporting Information). Eleven miRNAs exhibited the differential expression of at least fourfold, with nine of these miRNAs displaying upregulation (Figure 1M; Figure S2F and Table S1, Supporting Information). Even with a reduced threshold of a twofold change, the number of upregulated miRNAs was more than double that of the downregulated miRNAs (Table S1, Supporting Information). We first investigated the miRNAs whose expression differed by at least fourfold. The predicted target genes of these differentially expressed miRNAs were further subjected to the functional enrichment analyses and the gene ontology (GO) terms revealed an enrichment of several biological processes associated with lipid and glucose homeostasis, insulin signaling, and cell cycle transition (Figure 1N). Kyoto Encyclopedia of Genes and Genomes (KEGG) pathway analysis revealed significant enrichment in many pathways linked to glucolipid metabolism, such as PI3K‐Akt signaling, insulin signaling, IR, AMPK signaling, and nonalcoholic fatty liver disease (Figure 1O). The predicted network between miRNAs and their putative target genes‐associated KEGG pathway further highlighted the dominance of the PI3K signaling (Figure 1P,Q). In summary, the differentially expressed miRNAs in sperm may be involved in the regulation of glucose and lipid metabolism.

### 2.2 Lipid Metabolic Reprogramming‐Regulated Pparg–Dicer1 Axis during Spermatogenesis Was Implicated in the Upregulation of Sperm miRNAs in BPA‐Exposed Male Mice

**Figure 2:**
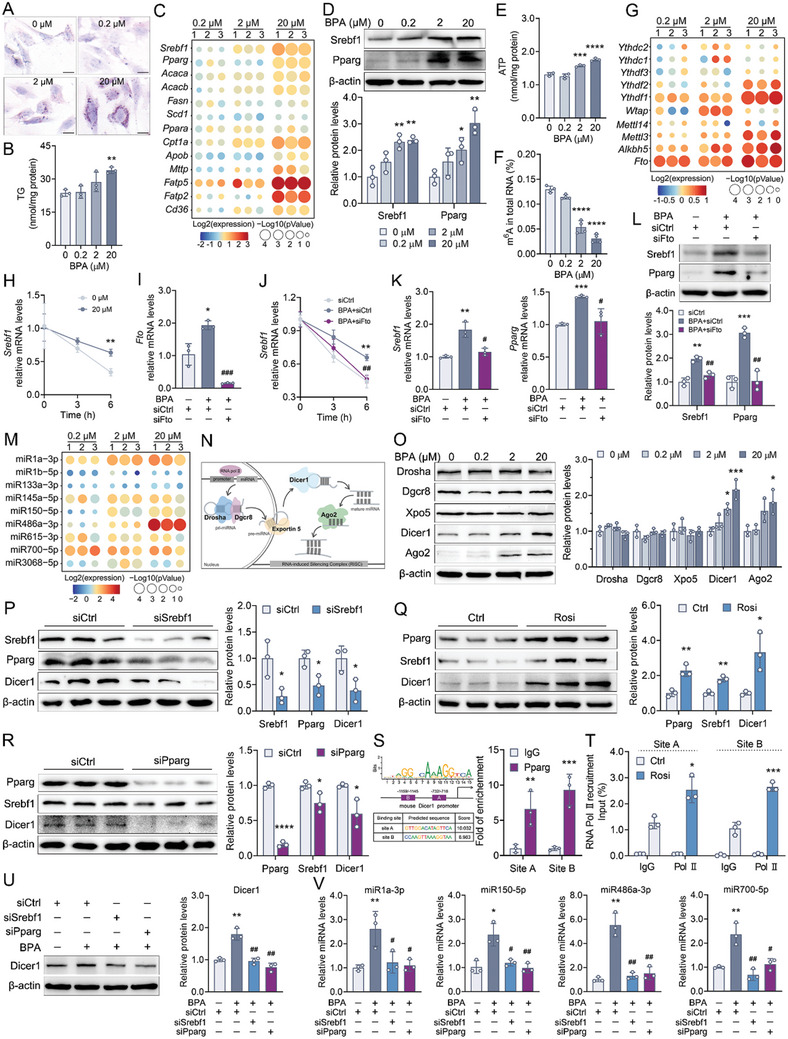
The role of Srebf1‐Pparg‐Dicer1 axis in the upregulation of sperm miRNAs. The GC‐2spd cells were treated with 0.2, 2 or 20 µm BPA for 48 h. A) Representative Oil Red O staining of lipids. B) The cellular TG content expressed as nmol/mg protein. C) The mRNA expression of genes related to lipid synthesis, uptake, oxidation, and secretion. D) The protein levels of Srebf1 and Pparg. Data were normalized to β‐actin. E) ATP content normalized to the cellular protein concentration. F) Global m^6^A level of total mRNA. G) The mRNA expression of m^6^A reader, writer, and eraser genes. (C,G) The quantitative results were normalized by β‐actin, and the data were presented as the log2‐transformed fold change. Circle size represented log10‐transformed *p*‐value, with larger circles representing smaller *p*‐values. H) The *Srebf1* mRNA decay assays. The GC‐2spd cells were treated with 20 µm BPA for 48 h, followed by actinomycin D (5 µg mL^−1^) treatment for 0, 3, or 6 h. The RNAs were then harvested, and the expression level of *Srebf1* mRNA was quantified by RT‐qPCR and normalized to β‐actin. I) The mRNA expression of *Fto* in GC‐2spd cells exposed to 20 µm BPA after Fto‐siRNA treatment. J) The role of Fto in stabilizing *Srebf1* mRNA in the BPA‐exposed cells. The GC‐2spd cells were cotreated with 20 µm BPA and 50 nm siFto for 48 h, after which mRNA decay assays were conducted. K,L) The role of Fto inhibition on the mRNA (K) and protein (L) expression of Srebf1 and Pparg. Data were normalized to β‐actin. The GC‐2spd cells were cotreated with 20 µm BPA and 50 nm siFto for 48 h. M) The expression of 13 upregulated miRNAs identified in sperm. U6 RNA served as the loading control. The data were presented as the log2‐transformed fold change. Circle size represented log10‐transformed *p*‐value, with larger circles representing smaller *p‐*values. N) The diagram of miRNA biogenesis pathway. O) The expression of key proteins in the miRNA biogenesis pathway. P) Effect of Srebf1 inhibition on the Pparg and Dicer1. The GC‐2spd cells were transfected 50 nm Srebf1 siRNA for 48 h. Q,R) The effect of Pparg overexpression (Q) and inhibition (R) on the expression of Srebf1 and Dicer1. For overexpression of Pparg, GC‐2spd cells were treated with 10 µm of Rosiglitazone (a full agonist for Pparg), while inhibition was achieved by 50 nm of siPparg. S) ChIP analysis of Pparg binding to the Dicer1 promoter. The fold enrichment levels were calculated relative to the IgG control after normalization to the respective inputs. The left panel showed the potential PPRE binding sequences in the promoter of Dicer1. T) The recruitment of RNA Polymerase II (Pol II) on the Dicer1 promoter. The GC‐2psd cells were treated with rosiglitazone for 48 h followed by ChIP‐qPCR assay. IgG was used as a control. Results were represented as the percentage of input (% of input). U) The protein expression of Dicer1. V) Effects of Srebf1 or Pparg inhibition on the BPA‐mediated upregulation of several miRNAs. U6 RNA served as the loading control. For T and U, GC‐2spd cells were treated with 20 µm BPA for 48 h and cotransfected with 50 nm of siSrebf1, 50 nm siPparg, or corresponding control siRNA (siCtrl) as indicated. All protein expression was quantified and normalized to β‐actin. *n* = 3 independent experiments. Data were presented as the mean ± SD. ^*^ *p* < 0.05, ^**^ *p* < 0.01, ^***^ *p* < 0.001, ^****^ *p* < 0.0001, versus unexposed controls. ^#^ *p* < 0.05, ^##^ *p* < 0.01, BPA and siFto (or siSrebf1, or siPparg) cotreated cells versus BPA and siCtrl cotreated cells.

A mouse spermatocyte cell line, GC‐2spd, was used to investigate how BPA elevated a large diversity of miRNAs in the sperm. Exposure of GC‐2spd cells to BPA led to a dose‐ and time‐dependent reduction in cell viability, and we selected BPA concentrations of 0.2, 2, and 20 µm, which maintained at least 80% cell viability after 48 h of exposure for subsequent experiments (Figure S3A, Supporting Information). **Figure** **2**A,B showed that the amount of lipid droplets and TG contents were increased in the 20 µm BPA‐treated GC‐2spd cells. Several genes involved in lipid metabolism, including de novo lipid synthesis, uptake, oxidation, and secretion, were also differentially expressed in the GC‐2spd cells treated with 2 and 20 µm BPA (Figure 2C). Moreover, the protein expression of Srebf1 and Pparg, which are critical lipogenic transcription factors, were increased in 2 and 20 µm BPA‐treated GC‐2spd cells (Figure 2D). The increased ATP production observed in these cells (Figure 2E) further indicated an enhancement in fatty acid oxidation, likely serving as a compensatory mechanism in response to lipid accumulation. BPA triggered lipid metabolic reprogramming in the spermatocytes.

As shown in Figure 2F, the overall level of N6‐methyladenosine (m6A) methylation was decreased in GC‐2spd cells treated with 2 and 20 µm BPA. The expression of methyltransferases (writers), demethylase (erasers), and methyl‐binding proteins (readers) was profiled and BPA increased *Fto*, *Alkbh5*, *Mettl3, Ythdf1*, and *Ythdf2* (Figure 2G)*. Fto* has been previously reported to promote hepatic lipogenesis by stabilizing *Srebf1* mRNA.^[^ ^15^ ^]^ Here, the mRNA stability of *Srebf1* in GC‐2spd cells treated with 20 µm BPA was also higher than in the untreated control cells (Figure 2H). Therefore, we knocked down Fto using siRNA in 20 µm BPA‐treated GC‐2spd cells to examine whether Fto expression influenced the mRNA stability of *Srebf1* and the expression levels of Srebf1 and Pparg (Figure 2I). Figure 2J–L showed that the inhibition of Fto abolished the increase in *Srebf1* mRNA stability in the 20 µm‐BPA exposed GC‐2spd cells and reduced mRNA and protein levels of Srebf1 and Pparg. Additionally, the mRNA expression of *Alkbh5*, *Mettl3*, *Ythdf1*, and *Ythdf2* remained unchanged after Fto silencing (Figure S3B, Supporting Information), suggesting that their upregulation might be secondary to Fto expression. BPA upregulated Srebf1 expression in spermatocytes (Figure 2C,D) and mature sperms (Figure S1F, Supporting Information) by stabilizing its mRNA, which may be related to the elevated *Fto* expression.

In line with the sperm miRNAs profile data, Figure 2M showed that the expression of miRNAs, including miR1a‐3p, miR145a‐5p, miR150‐5p, miR486a‐3p, miR‐700‐5p, and miR3068‐5p, was still elevated in the BPA‐treated GC‐2spd cells. We investigated whether BPA‐induced miRNAs upregulation could be the result of altered expression of key proteins in the miRNAs biogenesis pathway (Figure 2N) and found that the pre‐miRNA processing ribonuclease Dicer1 and the RNA‐induced silencing complex (RISC) endonuclease Ago2 were increased by BPA (Figure 2O; Figure S3C, Supporting Information). Figure 2P–R further illustrated the reciprocal positive regulatory loop among Srebf1, Pparg, and Dicer1 in the GC‐2spd cells. More importantly, the promoter region of Dicer1 was found to contain two Pparg response elements (PPREs). Utilizing CHIP‐qPCR, we confirmed that Pparg regulated Dicer1 expression by binding to PPREs within the Dicer1 promoter (Figure 2S). Moreover, in the Pparg‐overexpressing GC‐2spd cells, concomitant increase in RNA Polymerase II (Pol II) binding was also observed at the Dicer1 promoter upon Pparg binding (Figure 2T). This observation suggested that the elevated expression of Dicer1 resulted from de novo transcription. That was, Pparg bound to the PPREs in the Dicer1 promoter region, facilitating the recruitment of Pol II to the site and consequently enhancing the transcriptional activation of Dicer1. The inhibition of either Srebf1 or Pparg effectively abrogated BPA‐induced Dicer1 upregulation, leading to a reduction in the expression levels of miR1a‐3p, miR150‐5p, miR486a‐3p, and miR700‐5p (Figure 2U,V). Notably, the overexpression of Pparg, induced by the administration of its agonist rosiglitazone, led to the partial restoration of Dicer1 levels, which were downregulated as a consequence of Srebf1 depletion in BPA‐exposed GC‐2spd cells (Figure S3D, Supporting Information). Pparg likely served as a downstream effector in this regulatory pathway. In summary, Srebf1‐Pparg signaling was activated during BPA‐induced lipid reprogramming in the spermatocytes, which led to the upregulation of Dicer1, culminating in increased expression of numerous sperm miRNAs.

### 2.3 Paternal Transmission of BPA‐Induced MetS‐Like Symptoms to Adult Offspring

**Figure 3:**
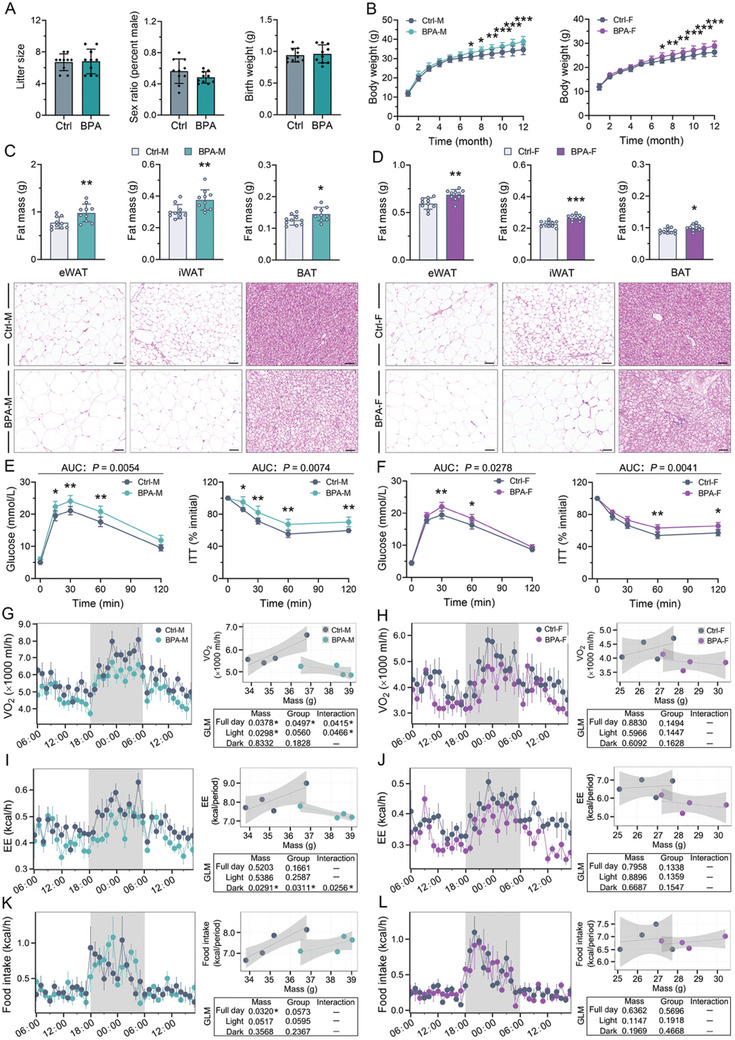
Paternal exposure to BPA triggered MetS‐like symptoms in the adult mice offspring. “M” meant male offspring and “F” meant female offspring. A) The litter size, gender ratio, and birth weight of neonatal offspring. B) The body‐weight‐change curves. C,D) The weight and histological images of visceral fat mass (eWAT), subcutaneous fat mass (iWAT), and brown fat mass (BAT) in male (C) and female offspring (D). E,F) The blood glucose during the GTT and ITT in male (E) and female offspring (F). The area under the blood glucose–time curve (AUC) was calculated and shown in Figure S7A,B of the Supporting Information. G–L) Metabolic flux analysis including O_2_ consumption (G,H), energy expenditure (I,J), and food intake (K,L). The time plots panel provided a visualization of individual metabolic parameters for the specified period, with shading indicating the dark and light photoperiods. Subsequently, the generalized linear model (GLM) was computed using metabolic parameters derived from the inputs on the left, with body weight incorporated as a covariate. The regression lines obtained from the GLM elucidated the associations among metabolic variables, including oxygen consumption, energy expenditure, or food intake and body weight, with the shaded region representing the standard error. The analytical table provided *p*‐values associated with group effects, as well as body weight and interaction effects, as necessitated by the GLM. *n* = 4–10 mice per group as indicated. Data were presented as the mean ± SD. ^*^ *p* < 0.05, ^**^ *p* < 0.01, ^***^ *p* < 0.001, versus corresponding controls.

Next, we investigated whether BPA‐induced MetS‐like symptoms in F0 male mice were inherited by the offspring. Control and BPA‐exposed male mice were mated with healthy females, and no apparent differences in litter size, sex ratio, or birth weight were observed between the paternal BPA‐exposed and control offspring (**Figure** **3**A). At the time of weaning (3 weeks of age), serum concentrations of soluble leptin receptor (sLepr) were decreased, whereas the free leptin index (FLI) was elevated in both male and female BPA‐exposed offspring (**Table** **1**). This early onset susceptibility to leptin resistance influenced the propensity of the BPA‐exposed offspring to develop obesity, and they showed a significant increase in body weight starting at 7 months of age (Figure 3B). At the age of 48 weeks, body fat mass and visceral (epididymal, eWAT) and subcutaneous (inguinal, iWAT) adipose tissue areas were increased in the paternal BPA‐exposed offspring (Figure 3C,D). These offspring exhibited typical signs of MetS that accompany obesity, including elevated levels of fasting serum TC, TG, FFA, insulin, and leptin, as well as reduced sLepr compared to the control offspring (Table 1). Furthermore, OGTT and ITT consistently revealed that glucose and insulin tolerance capacities in paternal BPA‐exposed offspring were significantly suppressed (Figure 3E,F). Paternal exposure to BPA did not affect the heart rate or blood pressure (BP) variability in the offspring (Table 1). In addition to the effects on glucose and lipid homeostasis, Figure 3C,D showed that the brown adipose tissue (BAT) mass and the proportion of enlarged adipocytes in BAT were increased in the paternal BPA‐exposed offspring. Paternally BPA‐exposed offspring, particularly males, also demonstrated a tendency toward reduced metabolic rate, as evidenced by the decreased energy expenditure and oxygen consumption, despite maintaining comparable food intake levels (Figure 3G–L). Among the male offspring, a significant interaction effect was observed between BPA exposure and body weight (covariate) concerning full‐day oxygen consumption and energy expenditure during dark cycles (Figure 3G,I). The regression analysis of energy expenditure and oxygen consumption against body weight revealed that paternally BPA‐exposed offspring expended less energy per unit increase in body weight, whereas control offspring expended more energy per unit increase (Figure 3G–J). Collectively, these findings suggested that paternal exposure to BPA induced early‐life leptin resistance and late‐onset MetS in the offspring, and no significant sex differences were observed in such father‐to‐offspring transmission.

**Table 1:** The serum biochemistry parameters, blood pressure, and heart rate in 3‐week‐old and 48‐week‐old offspring.

	3 weeks				48 weeks			
	Male		Female		Male		Female	
	Ctrl	BPA	Ctrl	BPA	Ctrl	BPA	Ctrl	BPA
Glucose [mmol L^−1^]	4.50 ± 0.55	4.25 ± 0.43	4.55 ± 0.45	4.32 ± 0.40	5.01 ± 0.53	5.83 ± 0.99^*^	4.28 ± 0.36	4.52 ± 0.48
Insulin [ng mL^−1^]	0.36 ± 0.08	0.38 ± 0.10	0.35 ± 0.08	0.34 ± 0.06	0.52 ± 0.13	0.85 ± 0.26^**^	0.44 ± 0.13	0.61 ± 0.14^*^
HOMA‐IR	1.52 ± 0.26	1.53 ± 0.35	1.50 ± 0.35	1.39 ± 0.26	2.45 ± 0.57	4.79 ± 1.83^**^	1.82 ± 0.62	2.60 ± 0.51^**^
Leptin [ng mL^−1^]	0.37 ± 0.08	0.39 ± 0.09	0.30 ± 0.07	0.33 ± 0.08	1.37 ± 0.23	3.33 ± 1.31^***^	1.18 ± 0.24	2.77 ± 1.25^**^
sLepr [ng mL^−1^]	3.83 ± 0.35	3.41 ± 0.38^*^	3.47 ± 0.43	3.09 ± 0.32^*^	1.76 ± 0.15	1.22 ± 0.14^****^	1.45 ± 0.19	1.11 ± 0.20^**^
FLI	0.10 ± 0.02	0.12 ± 0.02^*^	0.09 ± 0.02	0.11 ± 0.03^*^	0.78 ± 0.13	2.77 ± 1.15^****^	0.81 ± 0.09	2.46 ± 0.85^****^
TC [mmol L^−1^]	1.59 ± 0.14	1.59 ± 0.16	1.59 ± 0.20	1.57 ± 0.22	2.08 ± 0.26	2.41 ± 0.33^*^	1.53 ± 0.23	1.59 ± 0.19
TG [mmol L^−1^]	0.92 ± 0.12	0.92 ± 0.12	0.84 ± 0.16	0.85 ± 0.17	1.09 ± 0.19	1.45 ± 0.28^**^	1.04 ± 0.16	1.29 ± 0.26^*^
FFA [mmol L^−1^]	0.20 ± 0.03	0.19 ± 0.02	0.18 ± 0.03	0.18 ± 0.02	0.26 ± 0.08	0.42 ± 0.07^***^	0.24 ± 0.06	0.33 ± 0.06^**^
SBP [mmHg]	–	–	–	–	98.60 ± 9.4	101.6 ± 9.6	105.2 ± 8.8	106.8 ± 7.3
DBP [mmHg]	–	–	–	–	83.80± 8.2	85.8 ± 8.0	90.4 ± 7.0	91.8 ± 3.5
Heart rate [bpm]	–	–	–	–	492.20 ± 15.7	498.0 ± 27.0	539.2 ± 18.3	534.0 ± 28.9

FLI, free leptin index (the ratio of leptin to soluble leptin receptor concentrations). SBP, systolic blood pressure; DBP, diastolic blood pressure. ^*^ *p* < 0.05, ^**^ *p* < 0.01, ^***^ *p* < 0.001, ^****^ *p* < 0.0001, versus corresponding control offspring.

### 2.4 Multiple Upregulated Sperm miRNAs Were Inherited and Cooperatively Suppressed the Hepatic Lepr in the Paternal BPA‐Exposed Offspring

**Figure 4:**
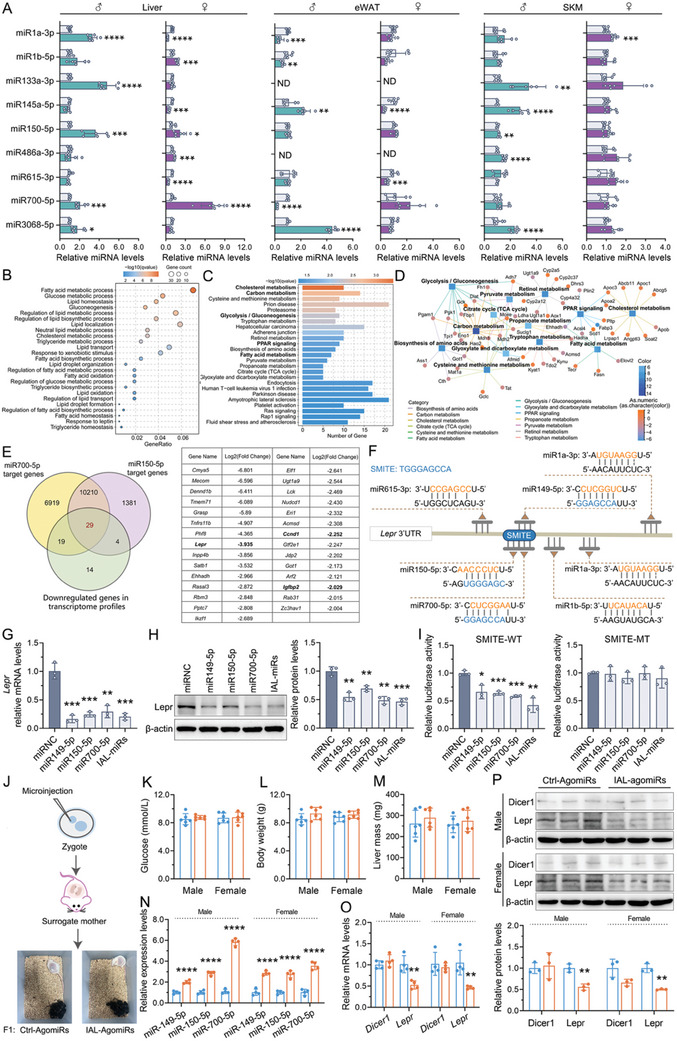
Inheritance testing and target gene screening of BPA‐upregulated miRNAs in paternal spermatozoa. A) The expression of sperm miRNAs in the metabolic organs (liver, skeletal muscle, and adipose tissue) in offspring. U6 was used as a loading control. B–D) Transcriptome analysis of hepatic gene expression profile in the male offspring. The PCA plot, the volcano plots, and the cluster heat map of the differential genes were shown in Figure S5A–C of the Supporting Information. B) The GO‐BP pathway related to glucose and lipid metabolism based on GO analysis in the liver transcriptome data. The top 25 enriched GO‐BP, GO‐CC, and GO‐MF terms were shown in Figure S5D–F of the Supporting Information. Color bars, the log (*q*‐value) of enrichment; Gene count, differential gene count in the indicated pathway; GeneRatio, differential gene count in the indicated pathway versus total differential gene count. C) The KEGG pathway enrichment analysis of the liver transcriptome data. Pathways involved in glucose and lipid metabolism were considered as hub pathways and bolded. D) The interaction network of differentially expressed genes mapped on the hub KEGG pathways. The square represented the hub KEGG pathways and the circle represented the differentially expressed genes. E) Venn diagram depicting the number of genes that overlap among miR700‐5p target genes, miR150‐5p target genes, and the downregulated genes (defined by a fold change ≤ −2 and an adjusted *p*‐value ≤0.05) identified in the liver transcriptomic analysis. The name and fold changes in the expression of 29 overlapped genes were summarized in the table on the right. F) The putative miRs binding sites in the 3′UTR of Lepr. G–I) AML12 cells were transfected with the single miR149‐5p mimic, miR150‐5p mimic, miR700‐5p mimic, mixture‐mimics (IAL‐miRs), or control mimic for 48 h. (G,H) The mRNA (G) and protein (H) expression of Lepr. Expression levels were normalized to β‐actin. (I) The relative luciferase activity in AML12 cells transfected with reporter vector containing wild (SMITE‐WT) or mutated (SMITE‐MT) “SMITE” binding sites. J–P) The impact of IAL‐AgomiRs overexpression in zygotes on offspring. (J) Schematic diagram of microinjection and the experimental procedure. IAL‐AgomiRs and corresponding Ctrl‐AgomiRs were microinjected into zygotes, which were subsequently transferred to surrogate mothers. The resulting offspring were kept for 14 days after birth for the further experiments (K–P). (K) The body weight in offspring. (L) The random blood glucose in offspring. (M) The liver weight in offspring. (N) The expression of miR149‐5p, miR150‐5p, and miR700‐5p in the liver of offspring. U6 RNA served as the loading control. (O,P) The mRNA (O) and protein (P) expression of Lepr and Dicer1 in the liver of offspring. β‐actin was used as an internal reference. (A–E) *n* = 3–6 mice per group as indicated; (G–I) *n* = 3 independent experiments per group; and (J–P) *n* = 3–6 mice from different litters/group as indicated. Data were presented as the mean ± SD. ^*^ *p* < 0.05, ^**^ *p* < 0.01, ^***^ *p* < 0.001, versus corresponding controls.

To investigate whether increased miRNAs in the sperm of F0 males could mediate the intergenerational inheritance of BPA‐induced MetS, we evaluated the effect of paternal BPA exposure on the expression of these miRNAs in the primary organs responsible for the regulation of glucose and lipid metabolism in the body, specifically in the liver, skeletal muscle, and adipose tissue in the progeny. **Figure** **4**A showed that miR150‐5p and miR700‐5p were highly expressed in the liver of both male and female offspring. Although miR1a‐3p, miR133‐3p, and miR3068‐5p expression were also increased in the liver of male offspring, they were downregulated and remained unaltered in the female offspring, respectively. In contrast, miR1b‐5p expression was increased in the liver of female offspring but not in males. Furthermore, there was no discernible pattern in the expression of all these sperm miRNAs in the skeletal muscle and adipose tissue of offspring of different sexes. We proposed that only the upregulation of miR150‐5p and miR700‐5p in spermatozoa could be reliably and consistently transmitted to the offspring and that the liver served as the primary target organ for these two miRNAs in the regulation of glycolipid metabolism. In addition, there were no changes in either mRNA or protein levels of Dicer1 in the liver of paternal BPA‐exposed offspring (Figure S4, Supporting Information). Consequently, the elevated expression of miR150‐5p and miR700‐5p in the liver of these offspring was attributed to paternal sperm transmission rather than direct regulation by Dicer1.

We performed a transcriptome analysis to identify the target genes of miR150‐5p and miR700‐5p in the liver of the male offspring. PCA analysis revealed that the transcriptome pattern of liver in paternally BPA‐exposed mice segregated from those of control offspring (Figure S5A, Supporting Information). The differential analysis volcano plot and heat map, presented in Figure S5B,C of the Supporting Information, indicated that 110 genes were upregulated and 66 genes were downregulated, with a greater than fourfold change. To further identify the main biological function in which the differentially expressed genes (DEGs) were involved, GO and KEGG enrichment analyses were performed. GO analysis revealed the enrichment of biological processes involved in fatty acid metabolism, glucose metabolism, lipid metabolism, and response to leptin (Figure 4B). Among the top 25 significantly enriched KEGG pathways, 5 were associated with the regulation of glucose and lipid homeostasis. These pathways included cholesterol metabolism, carbon metabolism, glycolysis/gluconeogenesis, PPAR signaling, and fatty acid metabolism (Figure 4C,D).

Next, target genes associated with both miR150‐5p and miR700‐5p were screened using the miRWalk database. These target genes were then cross‐referenced with DEGs that had been previously identified in the liver transcriptomes of male offspring. As illustrated in Figure 4E, the intersection of the three gene sets yielded a total of 29 overlapping genes. In this gene set, we found that Lepr 3′UTR contained multiple predicted binding sites for miRNAs that were upregulated within F0 male spermatozoa, including miR1a‐3p, miR1b‐5p, miR149‐5p, miR150‐5p, miR615‐3p, and miR700‐5p. More intriguingly, the binding sites on the *Lepr* 3′UTR for the miR149‐5p, miR150‐5p, and miR700‐5p were in proximity to each other, sharing a 9‐base pair region (905–913: TGGGAGCCA) (Figure 4F). Despite its absence from the 4‐fold differential gene expression list, BPA elicited a 3.1‐fold increase in the expression of miR149‐5p within the sperm of F0 male progeny (Table S1, Supporting Information). miR149‐5p expression was also increased in the liver of paternal BPA‐exposed offspring (Figure S6, Supporting Information). In the normal alpha mouse liver 12 (AML12) cells, the overexpression of miR149‐5p, miR150‐5p, or miR700‐5p, either individually or in combination, effectively downregulated Lepr (Figure 4G,H). The luciferase reporter assay results in Figure 4I further confirmed that miR149‐5p, miR150‐5p, and miR700‐5p acted as negative coregulators of Lepr through direct binding to this 9‐bp region in its 3′UTR. We, therefore, termed this region as “SMITE”, an acronym for “Several MiRNAs Targeting Elements”. The term “SMITE” denotes a powerful strike or knock, and we sought to employ this word to vividly illustrate that multiple inherited miRNAs significantly suppressed hepatic Lepr expression via binding to this 9‐bp region. miR149‐5p, miR150‐5p, and miR700‐5p were also defined as inherited anti‐Lepr miRNAs (IAL‐miRs). Jointly, IAL‐miRs bound to the “SMITE” region within 3′UTR of Lepr, thereby smiting its expression.

To substantiate the hypothesis that sperm IAL‐miRs functioned as molecular messengers, facilitating the intergenerational transmission of MetS by regulating hepatic Lepr signaling, AgomiRs that mimicked the three IAL‐miRs (miR149‐5p, miR150‐5p, and miR700‐5p) were injected into normal zygotes (Figure 4J). Subsequently, we conducted a comparative analysis of the differences between offspring born to zygotes injected with IAL‐AgomiRs and Ctrl‐AgomiRs. There were no significant treatment effects related to litter size and percentage of males. At 14 days of age, no differences in body weight, random blood glucose, or liver weight were observed between the groups (Figure 4K–M). The expression of miR149‐5p, miR150‐5p, and miR700‐5p was increased in the liver of IAL‐AgomiRs offspring (Figure 4N), but the expression of Dicer1 did not differ between offspring in the two groups. The upregulation of IAL‐miRs was also concomitant with the suppression of both mRNA and protein expression of Lepr in the liver of the IAL‐AgomiRs offspring (Figure 4O,P). No sex‐related differences were observed between the groups. Therefore, it is highly probable that the elevated levels of IAL‐miRs in the sperm of the F0 generation mice will result in leptin resistance and MetS in their offspring later in life through the hepatic IAL‐miRs‐Lepr pathway, akin to the phenotypes observed in offspring paternally exposed to BPA.

### 2.5 The Involvement of the Hepatic IAL‐miRs–Lepr Axis in Ectopic Lipid Accumulation and the Inhibition of Insulin Signaling in Paternal BPA‐Exposed Offspring

**Figure 5:**
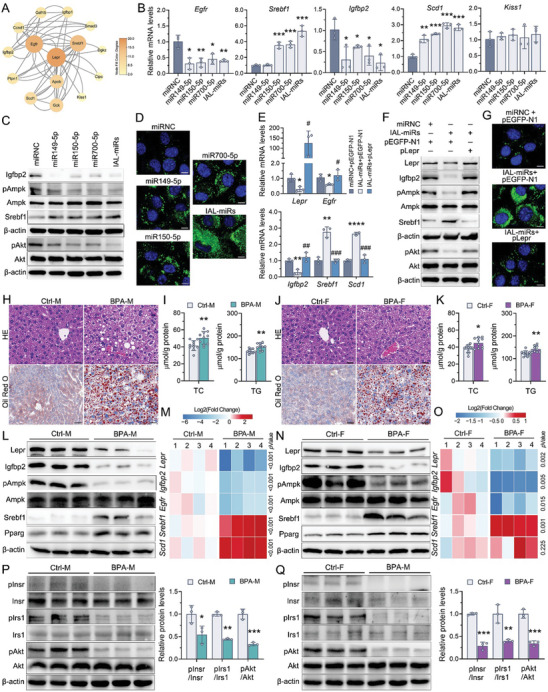
The IAL‐miRs–Lepr axis regulated hepatic lipid homeostasis and insulin signal in the paternal BPA‐exposed offspring. A) Lepr‐focused regulatory network. The node size indicated the closeness centrality in the network, and node color represented the degree of connectivity. B,E) The mRNA expression of hub genes in the Lepr‐focused regulatory network. β‐actin served as a reference gene. C,F) The expression of the downstream effector proteins involved in Lepr‐mediated regulation of glucose and lipid metabolism. The Akt phosphorylation was detected in the treated AML12 cells stimulated with 100 nm insulin for 15 min before harvesting. β‐actin served as a reference gene. The quantification protein bands were shown in Figures S8E and S9B of the Supporting Information. D,G) Lipid droplets labeled with BODIPY. The quantification of BODIPY‐positive cells was shown in Figures S8F and S9C of the Supporting Information. (B–D) The AML12 cells were transfected with the single miR149‐5p mimic, miR150‐5p mimic, miR700‐5p mimic, mixture‐mimics (IAL‐miRs), or control mimic (miRNC) for 48 h; and (E–G) the AML12 cells were cotransfected with the mixture‐mimics (IAL‐miRs), pEGFP‐Lepr (pLepr) or corresponding controls for 48 h as indicated. H,J) Representative images of HE‐ and Oil Red O‐stained liver sections in the male (H) and female offspring (J). I,K) The TC and TG contents in the liver of male (J) and female offspring (K). L–O) Validation of the protein (L,N) and mRNA (M,O) expression of genes regulated by Lepr in the liver of male (L,M) and female offspring (N,O). β‐actin was used as an internal reference. The quantification data of protein expression were shown in Figure S7C,D of the Supporting Information. P,Q) Analysis of insulin signal pathway in the liver of male (P) and female offspring (Q). The mice were fasted overnight, given an intraperitoneal insulin injection (1.0 IU kg^−1^), and sacrificed after 10 min to prepare liver protein extracts. β‐actin served as a reference gene. (B–G) *n* = 3 independent experiments/group; (L–Q) *n* = 3–4 mice per group as indicated. Data were presented as the mean ± SD. ^*^ *p* < 0.05, ^**^ *p* < 0.01, ^***^ *p* < 0.001, versus corresponding control offspring, or AML12 cells transfected with miRNC. ^#^ *p* < 0.05, ^##^ *p* < 0.01, ^###^ *p* < 0.001, AML12 cells cotransfected IAL‐miRs mimics and pEGFP‐N1 versus AML12 cells cotransfected IAL‐miRs mimics and pLepr.

To elucidate the regulatory mechanisms underlying Lepr, a gene list was derived from the intersection of differential genes in the liver transcriptome and the genes that directly interact with Lepr and lgfbp2. A novel new protein–protein interaction (PPI) network was constructed and visualized using the String online platform. Subsequently, the “Degree” algorithm in Cytoscape was employed to prioritize and assess the significance of genes within the PPI networks (**Figure** **5**A). Using the constructed networks, we validated several principal genes implicated in the IAL‐miRs–Lepr axis‐dependent regulation. We overexpressed IAL‐miRs, both individually and in combination, in AML12 cells. As demonstrated in Figure 5B,C, the overexpression of IAL‐miRs, whether in conjunction or individually, increased the expression of Srebf1 and Scd1 while simultaneously decreasing the expression of Igfbp2 and Egfr. Moreover, the overexpression of IAL‐miRs suppressed the phosphorylation of Akt and Ampk in the AML12 cells (Figure 5C). Consistent with these molecular changes, Figure 5D showed that IAL‐miRs promoted lipid accumulation, as evaluated by Bodipy staining. Hepatic Lepr overexpression effectively ameliorated both the steatosis and reduced insulin sensitivity induced by the IAL‐miRs upregulation (Figure 5E–G). These in vitro results were further corroborated by the metabolic phenotype detected in the liver of paternal BPA‐exposed offspring. In addition to steatosis (Figure 5H–K), paternal exposure to BPA led to a decrease in expression of Lepr, Igfbp2, and pAmpk, and upregulation of Srebf1 and Pparg (Figure 5L–O) in the liver of both male and female offspring. Dysregulation of the insulin‐stimulated Insr–Irs1–Akt cascade was also observed in the liver of both paternal BPA‐exposed offspring (Figure 5P,Q). Consequently, the sperm‐mediated IAL‐miRs–Lepr axis can be recognized as a critical signaling event that triggered the intergenerational transmission of BPA‐induced MetS.

### 2.6 Paternal Exposure to BPA Accelerated Hepatic Senescence in the Offspring

**Figure 6:**
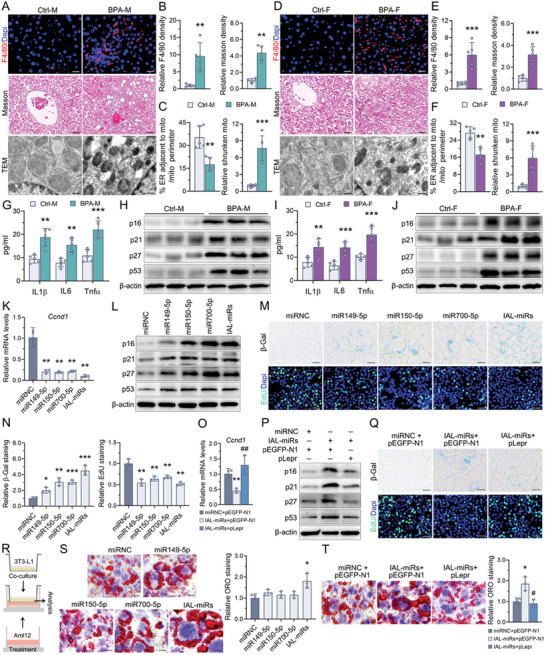
Paternal exposure to BPA accelerated hepatic senescence in the offspring. A–F) Representative images of the immunofluorescence staining for F4/80 (F4/80^+^ is red and Dapi is blue) and Masson's trichrome staining (blue is collagen), and the TEM images in the liver of male (A–C) and female offspring (D–F). The quantitation data were shown in the right panel. G,I) Detection of the serum inflammatory factors in the male (G) and female (I) offspring. H,J) The protein expression of several cellular senescence markers in the male (H) and female (J) offspring. β‐actin was used as an internal reference. The quantification of protein bands was shown in Figure S7I,J of the Supporting Information. K–N) AML12 cells were transfected for 48 h with either a single miR149‐5p, miR150‐5p, miR700‐5p mimic, a mixture of mimics (IAL‐miRs), or a control mimic (miRNC). O–Q) AML12 cells were cotransfected for 48 h with the mixture‐mimics (IAL‐miRs) and pEGFP‐Lepr (pLepr) or their corresponding controls. (K,O) The mRNA expression of *Ccnd1* in AML12 cells. β‐actin was served as a reference gene. (L,P) The protein expression of cellular senescence markers in the AML12 cells. β‐actin was used as an internal reference. β‐actin was used as an internal reference. The quantification of protein bands was shown in Figures S8H and S9D of the Supporting Information. (M,N,Q) The senescence‐associated SA‐β‐gal and EdU fluorescence staining for proliferation. Quantification of data was shown in the 5N (Figure S9E,F, Supporting Information). R–T) An in vitro transwell coculture system to study the effect of IAL‐miRs–Lepr pathway‐regulated hepatocytes senescent on adipocyte differentiation. (R) Schema of the coculture model. The transfected AML12 cells were seeded as donor cells and cocultured with recipient 3T3‐L1 adipocytes in a transwell system. (S,T) The fat storage evaluated using Oil Red O staining in adipocytes cocultured with AML12 cells transfected with either IAL‐miRs mimics (S) or both IAL‐miRs mimics and pLepr (T). (A–J) *n* = 3–4 mice per group as indicated; (K–T) *n* = 3 independent experiments/group. Data were presented as the mean ± SD. ^*^ *p* < 0.05, ^**^ *p* < 0.01, ^***^ *p* < 0.001, versus corresponding control offspring, or AML12 cells transfected with control mimic. ^#^ *p* < 0.05, ^##^ *p* < 0.01, ^###^ *p* < 0.001, AML12 cells cotransfected IAL‐miRs mimics and pEGFP‐N1 versus AML12 cells cotransfected IAL‐miRs mimics and pLepr.

In addition to glucose and lipid abnormalities, the liver weights were increased in the paternal BPA‐exposed offspring (Figure S7E,F, Supporting Information). The F4/80 and Masson's trichrome staining indicated that inflammation and mild fibrosis were exhibited in the liver of the paternal BPA‐exposed offspring (**Figure** **6**A,B,D,E). TEM imaging showed that paternal exposure to BPA decreased the proportion of the length of contact sites for those that were less than 50 nm between the ER and mitochondria relative to the total mitochondrial perimeter, which is a hallmark of the diminished ER–mitochondria contacts. Moreover, the mitochondria in hepatocytes from paternal BPA‐exposed offspring were smaller (Figure 6A,C,D,F). These morphological alterations were positively correlated with liver injury and aging. Additionally, the levels of inflammatory cytokines Tnfα, IL6, and IL1β in the peripheral blood were higher in paternal BPA‐exposed offspring than in corresponding controls (Figure 6G,I). Decreased expression of Cyclin D1 (Ccnd1) expression, a known marker of cellular senescence, was also observed in the liver of both male and female paternal BPA‐exposed offspring (Figure S7G,H, Supporting Information). Given the presence of two “SMITE” sites located in the 3′ UTR of Ccnd1 (Figure 4E; Figure S8G, Supporting Information), and the observed negative regulation of Ccnd1 by IAL‐miRs in AML12 cells (Figure 6K), we hypothesized a potential connection between IAL‐miRs and the initiation of liver senescence. Indeed, Figure 6H,J showed that the expression of key proteins involved in the regulation of senescence, including p53, p16, p21, and p27, was increased in the liver of paternal BPA‐exposed offspring. Moreover, overexpression of IAL‐miRs in the AML12 cells also upregulated all these molecular markers of senescence, which in turn augmented SA‐β‐gal activity and diminished cellular proliferative activity (Figure 6L–N; Figure S8I, Supporting Information). In addition, simultaneous overexpression of IAL‐miRs and Lepr in AML12 cells prevented the downregulation of Ccnd1, proliferative arrest, and senescence induced by IAL‐miRs (Figure 6O–Q). Ccnd1 and Lepr are target genes that played significant roles in the induction of cellular senescence, which is regulated by IAL‐miRs. Finally, 3T3‐L1 preadipocytes were cocultured with the AML12 cells during differentiation using the Transwell system (Figure 6R). As depicted in Figure 6S, overexpression of IAL‐miRs in AML12 cells facilitated the differentiation of 3T3‐L1 preadipocytes into adipocytes and enhanced TG storage within adipocytes in the coculture environment. This modulatory effect was reversed when Lepr was concomitantly overexpressed in IAL‐miR‐overexpressing AML12 cells in a coculture system (Figure 6T). We hypothesized that IAL‐miRs–Lepr‐regulated senescent hepatocytes might facilitate obesity through a senescence‐associated secretory phenotype (SASP), which in turn exacerbates the development of MetS in paternal BPA‐exposed offspring. However, additional data and comprehensive analyses are required to substantiate this mechanistic hypothesis in future studies.

## 3 Discussion

The metabolic state of an organism is susceptible to environmental stimuli, and these cues can be sensed and transmitted to the offspring. The familial aggregation of MetS among twins, siblings, as well as parents, and offspring has been implicated in numerous epidemiological studies.^[^ ^16^ ^]^ As we know, the preconception period is a particularly susceptible window for exogenous environmental toxin exposure. A wide variety of paternal exposures have been reported to influence offspring phenotype in mammals.^[^ ^17^ ^]^ In this study, male C57BL/6 mice exposed to BPA (50 µg kg^−1^ day^−1^) for 21 weeks exhibited metabolic disorders, including obesity, IR, and hyperlipidemia. When BPA‐exposed mice were used as sires and mated with healthy females, their offspring also developed MetS during adulthood. There were no significant differences between the sexes.

In the previous studies, we demonstrated that exposure to BPA at a concentration of 50 µg kg^−1^ day^−1^ induced hepatic steatosis, IR, and impaired insulin secretion.^[^ ^2a–c^ ^]^ Furthermore, perinatal exposure to this dose of BPA was associated with increased body weight and disrupted glucose and lipid homeostasis in adult offspring.^[^ ^7a^ ^]^ To further investigate, contrast, and deepen our understanding of these effects, the present study employed this specific dosage. Recently, a study providing a global evaluation of human exposure to BPA indicated that the reported daily intake values worldwide varied from 3.00 × 10^−5^ to 6.56 µg kg^−1^ day^−1^.^[^ ^18^ ^]^ However, in rhesus monkeys and mice, the maximum unconjugated serum concentration of BPA, measured at 3.95 ng mL^−1^, was observed 1 h following an oral administration of 400 µg kg^−1^ of BPA, subsequently decreasing to ≈0.52 ng mL^−1^ after 24 h. Based on the findings of this study, it was estimated that achieving serum BPA concentrations in humans ranging from 0.3 to 4 ng mL^−1^ would require a minimum daily oral intake of BPA at 500 µg kg^−1^ day^−1^.^[^ ^19^ ^]^ A recent human biomonitoring study conducted in a densely industrialized area reported a 100% detection rate of BPA in human serum samples, with a mean serum concentration of 42.062 ng mL^−1^.^[^ ^20^ ^]^ These findings raise concerns that the current estimates of human BPA exposure levels might be significantly underestimated. The dose used in this study largely fell within the concentration ranges observed in human‐relevant exposure scenarios. Notably, the U.S. Environmental Protection Agency established a reference dose of 50 µg kg^−1^ day^−1^ to evaluate the safety of BPA exposure several years ago, while the European Food Safety Authority has recently reassessed the public health risks associated with BPA and proposed a 20 000‐fold reduction in the tolerable daily intake, setting it at 0.2 ng kg^−1^ day^−1^.^[^ ^21^ ^]^ Therefore, our concerns should focus on the effects of lower doses of BPA on glucose and lipid metabolism in future studies.

Mammalian sperm RNA is another source of hereditary paternal information beyond DNA. Spermatozoa contain a diverse range of small noncoding RNA subtypes (sncRNAs, <40 nt), including miRNAs, endogenous small interfering RNAs (endo‐siRNAs), germline‐specific PIWI‐interacting RNAs (piRNAs), and several cleavage products of noncoding RNAs, notably transfer RNAs (tRNAs) that are commonly referred to as tRNA fragments or “tRFs (transfer RNA fragments)”.^[^ ^22^ ^]^ Moreover, approximately half of sncRNAs present in the single‐cell embryos originate from the paternal sources.^[^ ^23^ ^]^ Sperm sncRNAs act as the crucial information carriers for transferring paternally acquired metabolic abnormalities to the offspring. For example, the transmission of metabolic phenotypes from obese, prediabetic F0 males to F1 and F2 was found to be associated with the inheritance of several RNA species, including tRFs and miRNAs, such as miR10.^[^ ^24^ ^]^ In another study, the expression levels of let‐7 family miRNAs were modified in the sperm of mice subjected to a protein‐restriction diet, consequently leading to metabolic alterations in the subsequent generation of mice.^[^ ^25^ ^]^ Microinjection of sperm RNA into normal zygotes generated a Western‐like diet‐induced phenotype in the resulting progenies.^[^ ^26^ ^]^ In this study, the total RNA extracted from the sperm of F0 mice was subjected to sncRNA sequencing, and the data indicated that mature miRNAs exhibited the highest relative abundance in sperm, ranging from 69% (control mice) to 79% (BPA‐exposed mice), followed by piRNAs at 7% to 10% (control mice), and rRNAs at 5% (BPA‐exposed mice) to 10% (control mice) (Figure S2B, Supporting Information). BPA mainly affected the expression patterns of miRNAs and piRNAs (Figure S2C,F, Supporting Information). Given that our previous studies had identified the susceptibility of miRNAs to BPA exposure,^[^ ^2a,c^ ^]^ this study first linked miRNAs to the intergenerational inheritance of MetS. Of the 1253 miRNAs identified in the mouse sperm, 13 miRNAs were differentially expressed fourfold or greater between BPA‐exposed and control mice. Altered miRNAs in sperm may reset the epigenetic state of the embryo by regulating target mRNA in the zygote and, consequently, lead to the intergenerational transmission of abnormal phenotypes. For instance, some miRNAs exhibited differential expression patterns in the stress‐exposed sperms, thereby targeting crucial nodes within the gene regulatory networks in early zygotes. These subsequent cascading reactions ultimately reprogramed the brain gene expression profile in the offspring.^[^ ^27^ ^]^ Notably, the upregulation of specific miRNAs in the sperm of adult mice subjected to traumatic stress was also observed in the hippocampus and hypothalamus of adult offspring, which are brain regions associated with the stress response.^[^ ^28^ ^]^ Paternal exposure to nicotine decreased miR15b levels in both the spermatozoa of F0 mice and the brains of F1 mice, leading to hyperactivity and reduced depression in the offspring.^[^ ^29^ ^]^ These studies proposed an additional mechanism for miRNAs‐based intergenerational transmission, indicating that the alterations in miRNAs introduced by spermatozoa can be reliably inherited and sustained in the somatic tissues of the offspring. According to the pathway enrichment analysis, we showed that the altered miRNAs in the sperm of BPA‐exposed mice were mainly predicted to target key nodes in the signaling pathways, including PI3K‐Akt, MAPK, mTOR, cAMP, calcium signaling or lipid, and atherosclerosis. Therefore, differentially expressed miRNAs can play a crucial role in regulating glucose homeostasis and lipid metabolism if they are transmitted to the primary organ responsible for the metabolism of their offspring.

In this study, a significant increase in the expression of miR149‐5p, miR150‐5p, and miR700‐5p was observed in the liver of both male and female progeny mice, which correlated with their expression in the sperm of BPA‐exposed F0 male mice. miR149‐5p has been recognized as a tumor suppressor owing to its capacity to inhibit migration and invasion in various types of tumor cells;^[^ ^30^ ^]^ however, studies have indicated that inhibition of miR149‐5p suppressed lipogenesis and reduced lipid droplet accumulation in both HepG2 and 3T3‐L1 cells.^[^ ^31^ ^]^ Nuclear miR150‐5p was found to facilitate hepatic lipid accumulation by targeting of RNA transcripts that overlap with the PLIN2 promoter.^[^ ^32^ ^]^ Diabetes‐induced‐miR150‐5p aggravated pyroptosis‐dependent skeletal loss and the inflammatory responses.^[^ ^33^ ^]^ Moreover, miR150‐5p knockout improved systemic metabolism and adipocytes in mice.^[^ ^34^ ^]^ As to miR700‐5p, several studies have reported that it participated in the regulation of smoking‐related interstitial fibrosis^[^ ^35^ ^]^ and bleomycin‐induced lung fibrosis;^[^ ^36^ ^]^ however, none has pointed it as a regulator of glucose homeostasis and lipid metabolism. Our study was the first to propose three inherited miRNAs synergistically inhibited Lepr via an overlapping “SMITE” region within the 3′UTR of Lepr. Notably, microinjection of the three synthetic miRNAs (Ago‐miR149‐5p, Ago‐miR150‐5p, and Ago‐miR700‐5p) into zygotes resulted in the suppression of hepatic Lepr expression in the offspring as early as 14 days of age, mirroring the phenotypes seen in paternal BPA‐exposed offspring. Thus, we proposed naming these sperm‐delivered miRNAs “inherited anti‐Lepr miRNAs (IAL‐miRs)”.

Leptin is an adipocyte‐derived hormone that transmits catabolic signals to the central nervous system (CNS) to regulate appetite and body energy expenditure. The biological effects of leptin are mediated by activation of the Lepr, which is expressed in both the CNS and peripheral tissues. In this study, commensurate with an increase in adipose mass and hyperinsulinemia, serum leptin levels were elevated in paternally BPA‐exposed offspring. In theory, the hepatic Lepr expression increases to generate soluble Lepr (sLepr) after stimulation by leptin, thereby governing the bioavailability and bioactivity of leptin.^[^ ^37^ ^]^ However, in the paternally BPA‐exposed offspring, the IAL‐miRs suppressed hepatic Lepr, and serum sLepR levels were also decreased. Defects in leptin activity are hallmarks of hepatic leptin resistance. sLepR plays a crucial role in regulating circulating leptin levels without increasing leptin transcription in adipose tissue.^[^ ^37^, ^38^ ^]^ The levels of sLepR were inversely correlated with obesity and altered glucose and lipid metabolism in humans.^[^ ^39^ ^]^ In this study, paternally BPA‐exposed offspring also exhibited similar food intake but lower energy expenditure when compared to the controls, suggesting that paternal exposure to BPA attenuated hepatic Lepr signaling and induced leptin resistance in the offspring. Moreover, we found that leptin resistance occurred before the development of significant changes in body weight and insulin sensitivity in paternally BPA‐exposed offspring as early as the third week of life, identifying it as an early factor in the pathogenesis of MetS.

On the one hand, the diminished sensitivity to leptin can potentially disrupt the regulation of the adipoinsular axis, thereby exacerbating hyperinsulinemia and IR.^[^ ^40^ ^]^ On the other hand, hepatic Lepr defect could induce weight gain, liver steatosis, and liver function damage independent of leptin/Lepr signaling from neurohumoral regulation governed by CNS.^[^ ^41^ ^]^ These metabolic abnormalities could be ameliorated by re‐expressing Lepr.^[^ ^42^ ^]^ In this study, Lepr was consistently downregulated in the liver of BPA‐exposed paternal generations and their progeny. Malfunctioning Lepr continued to deactivate Ampk by inhibiting its phosphorylation in the liver of paternally BPA‐exposed offspring. An immediate role of Ampk inactivation was to dephosphorylate Akt and to enhance the de novo synthesis of lipids through the activation of *Srebf1*, *Fasn*, and *Scd1*. We then focused on hepatic Lepr, constructed PPI networks, and identified the associated interaction partners implicated in the regulation of metabolic homeostasis within the pool of DEGs. In AML12 cells, the overexpression of IAL‐miRs resulted in the inhibition of Igfbp2 and Egfr as well as the elevation of Srebf1 and Scd1 within the PPI network. The regulatory effects of IAL‐miRs were counteracted by Lepr overexpression, thereby confirming that Lepr was a central upstream factor in the network.

Although no “SMITE” was identified in Igfbp2 3′UTR, we showed that it could be indirectly regulated by IAL‐miRs through Lepr. The expression of Igfbp2 was significantly decreased in the liver of paternally BPA‐exposed mice and in the IAL‐miRs‐overexpressing AML12 cells. Evidence indicated a correlation between low circulating Igfbp2 levels and metabolic dysfunction, including obesity, dyslipidemia, and IR, in both human and rodent models.^[^ ^43^ ^]^ Liver is the primary organ responsible for the production of Igfbp2.^[^ ^44^ ^]^ In HFD‐fed mice, hepatic Igfbp2 was downregulated and negatively associated with TG content. Lower hepatic Igfbp2 expression was negatively associated with liver fat deposition.^[^ ^43a^ ^]^ Consistently, hepatic overexpression of Igfbp2 reduced lipid accumulation and IR in the metabolism‐associated fatty liver disease phenotype in obese mice and in palmitic acid‐treated AML12 cells.^[^ ^45^ ^]^ Igfbp2 activated glucose metabolism and insulin pathway signaling through the Igfbp2‐Ampk signaling pathway,^[^ ^46^ ^]^ whereas Igfbp2 inhibition hindered the induction of Ampk activation.^[^ ^47^ ^]^ In this study, the PPI network of the Lepr‐related gene cluster also revealed a possible functional relationship between Lepr, Igfbp2, and Egfr, which exhibited consistent downregulation in the liver of paternally BPA‐exposed offspring. Overexpression of Lepr resulted in the elevation of Egfr in the IAL‐miRs overexpressing AML12 cells. Egfr is one of most highly expressed growth factors in the hepatocytes of the adult liver,^[^ ^48^ ^]^ whereas Igfbp2 formed a complex with Egfr, thereby regulating the nuclear Egfr signaling pathway in certain carcinoma cells.^[^ ^49^ ^]^ The ectopic expression of Egfr ameliorated hepatic lipid accumulation and reduced serum TC and TG levels in HFD‐fed mice.^[^ ^50^ ^]^ Conversely, knockdown of Egfr suppressed Akt signaling, which is known to regulate hepatic lipogenesis by modulating lipogenic transcriptional factors, such as Srebf1 and Pparg.^[^ ^51^ ^]^ In light of these points, we suggested that Lepr, either directly or indirectly through Igfbp2 and Egfr, regulated insulin sensitivity and lipid deposition in the liver of paternally BPA‐exposed offspring.

In this study, Pparg expression was increased in the paternal BPA‐exposed offspring. Pparg dysregulation was associated with substantial metabolic consequences and has been implicated in the pathogenesis of metabolic disorders, including obesity, IR, and type 2 diabetes. The regulation of Pparg constitutes a complex process involving multiple signaling pathways and coregulatory factors, and Pparg exhibits diverse functions across various tissues and cell subtypes, resulting in distinct physiological roles.^[^ ^52^ ^]^ As we known, Pparg is highly expressed in the adipose tissue, and the activation of Pparg exerts significant adipogenic and antidiabetic effects within adipocytes. Constitutively active Pparg facilitates adipocyte differentiation, enhances fatty acid uptake, and promotes fatty acid storage in lipid droplets, thereby reducing ectopic lipid deposition and improving systemic insulin sensitivity.^[^ ^53^ ^]^ Blocking anti‐Pparg activity of TAZ in adipocytes leads to reduced inflammation and the restoration of insulin sensitivity and glucose homeostasis.^[^ ^54^ ^]^ Furthermore, Pparg serves as a sensor that interacts with adiponectin and other molecules to mitigate IR.^[^ ^52b^ ^]^ In skeletal muscle, Pparg modulates glucose uptake via Glut4 and Irs1, and knockout of Pparg in mouse skeletal muscle leads to progressive IR.^[^ ^55^ ^]^ Beyond its insulin‐sensitizing benefits, it is crucial to acknowledge its adverse effects on other tissues. Overexpressing Pparg in pancreatic β‐cells led to increased islet cell apoptosis, reduced β‐cell mass, and exacerbated obesity‐induced glucose intolerance in mice.^[^ ^56^ ^]^ More importantly, numerous studies in animal models of obesity and diabetes have reported that increased hepatic expression of Pparg exacerbates hepatic lipid accumulation. Pparg increases the transcription of Srebf1, which activates other adipogenic genes and promotes the conversion of pyruvate to fatty acids.^[^ ^57^ ^]^ Conversely, hepatocyte‐specific knockout of Pparg in leptin‐deficient mice ameliorated hepatic steatosis and exacerbated diabetic phenotypes.^[^ ^58^ ^]^ Several Pparg antagonists have also been reported to mitigate hyperglycemia and hyperinsulinemia and to reduce the plasma levels of Tnf in ob/ob mice.^[^ ^59^ ^]^ Taken together, the proadipogenic influence of Pparg in the liver exacerbates hepatic lipid accumulation and worsens IR. However, in adipocytes, Pparg facilitates the storage of excess lipids, thereby reducing lipid influx into the liver and restorting glucose homeostasis and insulin sensitivity. These findings are consistent with the observations, that IAL‐miRs upregulated Pparg and suppressed Akt insulin signaling in the liver of paternal BPA‐exposed offspring. These IAL‐miRs exhibited a tissue‐specific expression pattern in the offspring, which was not differentially expressed in adipose tissue and skeletal muscles, potentially diminishing the therapeutic efficacy of Pparg in addressing IR. Under the synergistic influence of chronic inflammation, paternal BPA‐exposed offspring exhibited IR and dysregulated lipid metabolism.

To the best of our knowledge, when the conserved epigenetic mechanisms, including miRNAs regulation, served as the underlying pathogenic mechanisms, it is anticipated that the resulting MetS phenotypes would demonstrate consistency across successive generations. Studies have shown that paternal exposure to environmental chemicals as well as factors such as diet and stress can induce metabolic disease phenotypes that persist across multiple generations. For example, paternal exposure to the endocrine disruptor di‐(2‐ethylhexyl) phthalate elicited glucose intolerance in female F2 offspring, possibly due to changes in the sperm tsRNA/rsRNA landscape.^[^ ^60^ ^]^ Similarly, adult F1 offspring sired by F0 fathers subjected to a high‐fat diet exhibited glucose intolerance, a phenotype that persisted in the F2 generation.^[^ ^61^ ^]^ Moreover, diet‐induced obesity in male mice led to alterations in small noncoding RNAs within the sperm, thereby predisposing male progeny in subsequent generations to obesity.^[^ ^62^ ^]^ For BPA, exposure during pregnancy resulted in obesity in the F2 offspring, attributable to increased food consumption, and this trait persisted up to the F6 generation.^[^ ^63^ ^]^ However, few reports on whether paternal exposure to BPA causes transgenerational metabolic abnormalities. The present study was the first to show that sperm‐borne miRNAs, which were affected by paternal BPA exposure, facilitated the intergenerational transmission of MetS. However, we did not extend our monitoring to determine whether the alterations in sperm‐derived miRNAs observed in the F1 generation persisted into the F2 generation. A previous study indicated that females in a depression‐like model could pass depressive traits to the F1 generation, but not to F2, possibly because of the absence of miRNAs changes in F1 sperm.^[^ ^13^ ^]^ Consequently, the lack of F1 sperm miRNAs data hindered our ability to speculate on the transgenerational transmission effects of paternal BPA exposure, thus representing a limitation of this study. Despite these limitations, it has been showed that both maternal and paternal exposure to BPA contributed to impaired spermatogenesis, reduced sperm count, and alterations in the biochemical and epigenetic properties of sperm in the F1 (offspring), F2 (grand‐offspring), and potentially F3 (great‐grand‐offspring) generations.^[^ ^64^ ^]^ These transgenerational epigenetic modifications in spermatozoa have been linked to the hereditary transmission of certain diseases.^[^ ^65^ ^]^ As a result, the transgenerational effects of paternal BPA exposure should be a promising research area warranting further investigation.

Premature cellular senescence is a state of irreversible cell‐cycle arrest that plays a significant role in the progression of numerous chronic diseases.^[^ ^66^ ^]^ In this study, we found that the protein expression of cell senescence marker SA‐β‐gal and cell cycle‐related p53, p21, p16, and p27 were enhanced in the liver of offspring with MetS, suggesting paternal BPA exposure drove hepatocytes underwent functional senescence. In terms of molecular mechanisms, Igfbp2 exerts a significant influence on the processes of senescence and aging. Igfbp2 silencing in mouse lung epithelial cells increased P21 levels and SA‐β‐gal activity in response to profibrotic stimuli, while transgenic aged mice expressing human Igfbp2 diminished senescence and profibrotic SASPs, resulting in amelioration of bleomycin‐induced pulmonary fibrosis.^[^ ^67^ ^]^ Here, the AML12 cells overexpressing IAL‐miRs exhibited an increase in the number of SA‐β‐gal positive cells and a suppression of cell proliferation. However, Lepr overexpression rescued the senescence‐like phenotype in these cells induced by IAL‐miRs. Hepatic miRs–Lepr axis‐mediated downregulation of Igfbp2 appeared to be one of the main mechanisms contributing to the induction of senescence‐associated markers. Through transcriptome profiling, we also found that Ccnd1, a crucial regulator of the cell cycle progression, was decreased in the liver of paternally BPA‐exposed offspring. More intriguingly, Ccnd1 was a common target gene for IAL‐miRs, and similar to Lepr, the presence of “SMITE” was detected within its 3′UTR. On the other hand, it was recently reported that Igfbp2 deletion resulted in ablation of Ccnd1 and Ki‐67 in midlobular zone 2 hepatocytes,^[^ ^68^ ^]^ implying that IAL‐miRs regulated Ccnd1 not only via direct binding to “SMITE” but might also via Igfbp2. To date, the role of Ccnd1 in abnormal glucose and lipid metabolism‐associated liver senescence has been poorly investigated; however, considerable evidence suggests that Ccnd1 is essential for cancer cells to remain in the proliferative and nonsenescence stages. The Akt/Ccnd1/p21/p27 pathway was involved in the regulating the proliferation and migration of liver cancer cells.^[^ ^69^ ^]^ And p27 mediated the cell cycle arrest and cellular senescence in Ccnd1‐depleted breast cancer cells.^[^ ^70^ ^]^ Hence, apart from p21 signaling, IAL‐miRs suppressed Ccnd1 to facilitate p27 expression, which was proposed as an additional mechanism contributing to hepatocyte‐senescence in the offspring with MetS. Senescence of the liver tissue is likely to increase the production and secretion of proinflammatory SASPs, thereby leading to the emergence of local inflammatory alterations in the liver. Consequently, NAFLD progressed to nonalcoholic steatohepatitis in the paternally BPA‐exposed offspring. Release of SASPs such as IL6, Tnfa, and IL1β into the circulation drove paternally BPA‐exposed offspring to a chronic inflammatory state. This, in turn, resulted in the exacerbation of multiple MetS features, including adiposity, IR, and dyslipidaemia, in the offspring. The removal of senescent cells using a senolytic cocktail of dasatinib and quercetin reduced hepatic steatosis in ageing, obese, and diabetic mice.^[^ ^71^ ^]^ In cell culture systems, we validated that the cocultured of 3T3‐L1 cells with AML12 cells overexpressing IAL‐miRs increased the conversion of 3T3‐L1 preadipocyte differentiation into adipocytes. However, this enhancement was diminished when 3T3‐L1 cells were cocultured with AML12 cells that were co‐overexpressing of IAL‐miRs and Lepr. Lepr‐Igfbp2‐Ccnd1 signaling may be a novel target for the treatment of the hepatocyte‐specific senescence‐MetS phenotype.

In this study, we demonstrated a novel finding regarding the mechanism by which BPA altered sperm miRNA profiles using a mouse spermatocyte‐derived *GC*‐*2spd* cell line. In vitro administration of BPA elevated the lipid content and upregulated the essential transcription factor Srebf1, which is involved in lipid biosynthesis. BPA also elevated the expression of some lipogenic enzymes including *Fasn*, *Acaca*, *Acacb*, and *Scd1*. Lipid accumulation in nonadipose cells induces cellular dysfunction. Consistent with previous findings in hepatocytes with massive accumulation of lipids,^[^ ^2a^ ^]^ the m6A amounts in total RNA were decreased and the mRNA expression of m6A demethylase *Fto* was increased in BPA‐exposed *GC*‐*2spd* cells. The upregulation of *Fto* had the ability to stabilize *Srebp1f* mRNA, which was possibly one of the reasons for the high levels of Srebf1 in the BPA‐treated G*C*‐*2spd* cells. Additionally, another lipid metabolic gene Pparg was positively correlated with BPA exposure, and chromatin immunoprecipitation (ChIP) analysis confirmed that PPARg directly bound to the promoter region of Dicer1 to upregulate its expression. Dicer1 is an essential enzyme involved in miRNAs maturation. As we know, miRNA biogenesis is a well‐organized multistep process that involves several modulators, including Dgcr8, Drosha, Xpo5, Dicer1, and Ago2. Dysregulation of miRNAs biogenesis component has a substantial impact on the maturation process of miRNAs, leading to altered miRNAs expression patterns.^[^ ^72^ ^]^ Due to the upregulation of Dicer1 and Ago2, two fundamental constituents of the RISC, we observed a trend toward an overall increase in miRNAs in the BPA‐exposed GC‐2spd cells. The observed miRNAs expression trend in this study was consistent with the findings in the sperm of male mice exposed to BPA. Moreover, we further evaluated the impact of BPA exposure on the expression levels of both primary IAL‐miRs (pri‐IAL‐miRs) and precursor IAL‐miRs (pre‐IAL‐miRs) in the sperm of F0 male mice and GC‐2spd cells, and showed that BPA did not affect the levels of pri‐miR149‐5p, pri‐miR150‐5p, or pri‐miR700‐5p in sperm and spermatocyte, and no significant alterations were observed in the levels of their corresponding pre‐IAL‐miRs (Figure S10, Supporting Information). Therefore, the observed increase in mature IAL‐miRs in the sperm of BPA‐exposed mice was not attributable to enhanced transcriptional activity. Instead, we suggested that the upregulation of miRNAs induced by BPA treatment was mediated by Dicer1‐dependent pre‐miRNAs processing. The knockdown of Srebf1 or Pprag suppressed the BPA‐induced increase in Dicer1 and miRNAs in the GC‐2spd cells, whereas the overexpression of Pparg counteracted the Srebf1 knockdown‐mediated downregulation of Dicer1 and miRNAs. Pparg is a downstream effector of Srebf1 in regulating Dicer1. Taken together, BPA‐triggered lipid metabolic reprogramming in the spermatocytes is involved in the regulation of the sperm miRNA profiles via the Srebf1–Pparg–Dicer1 axis.

## 4 Conclusion

Exposure to BPA caused metabolic disorders in the parents, and such phenotypes were transmitted to the offspring, leading to the development of late‐onset MetS. During the intergenerational transmission, a set of miRNAs displayed consistent upregulation patterns in the sperm of F0 male mice exposed to BPA as well as in the liver of their offspring. The upregulation of most sperm miRNAs can be attributed to the influence of BPA on miRNA biogenesis via the Srebf1‐Pparg‐Dicer1 axis in spermatocytes. Hereditary miRNAs regulated hepatic glucolipid metabolism and senescence through a Lepr‐centric core network, which encompassed Igfbp2, Ccnd1, Egfr, and Ampk. Collectively, the present study revealed a novel mechanism underlying the intergenerational transmission of paternally acquired MetS.

## 5 Experimental Section

### Animals and Treatments

All animal experiments were approved by the guidelines of the Xiamen University Institutional Committee for the Care and Use of Laboratory Animals (XMULAC20200200). Adult male and female C57BL/6J mice were purchased from SLAC Laboratory Animal Center (Shanghai, China) and housed in the animal facility under specific pathogen‐free conditions with controlled room temperature (22 ± 2 °C), humidity (55 ± 5%), and 12:12 h light‐dark cycle. Mice were allowed ad libitum access to water and were randomly assigned to one of two groups: the control group, which was provided with a normal chow diet (NCD, 3616.0 kcal kg^−1^), and the BPA‐exposed‐group, which received an NCD supplemented with 0.5 µg g^−1^ of BPA. On the average daily consumption of a 2.5–3.0 g diet, the intake of BPA was estimated to be 50 µg kg^−1^ body weight per day. Following 21 weeks of BPA exposure, the male mice were mated to healthy virgin F0 females (Figure S1G, Supporting Information). Successful mating was judged by the presence of vaginal plugs the following morning. After mating, male F0 mice were removed and the gestational females were housed individually. At birth (Day 0), the offspring's gender was recorded and the body weight was measured. The litters were subsequently nurtured by their biological mothers until weaning (3 weeks of age). After weaning, litters were segregated according to sex and treatment. Two male and two female pups were selected randomly per litter (12 litters per group) and were raised until 48 weeks of age. Body weight was measured from week 3 to week 48. The offspring were anesthetized and sacrificed by decollation at 3 and 48 weeks of age, respectively. To mitigate the influence of litter effects, the experiments were conducted on offspring originating from distinct litters within each treatment group. The animal study flowchart is described in Figure 1A.

### Synthetic miRNAs Microinjection and Embryo Transfer

AgomiRs that specifically mimic endogenous IAL‐miRs and the corresponding AgomiRs control were synthesized by RiboBio (Guangzhou, China) and embryonic microinjections were performed. Briefly, equal amounts of three chemically synthetic AgomiRs (miR149‐5p, miR150‐5p, and miR700‐5p) were mixed and adjusted to a concentration of 2 ng µL^−1^ according to previous studies.^[^ ^13^, ^28^ ^]^ Then the AgomiRs mixture were microinjected into zygotes of the C57Bl/6J background using a Leica microinjection system. Microinjected zygotes were cultured overnight to the two‐cell stage and then were transferred to surrogate mother of the ICR background. The resultant offspring were raised for the subsequent experiments.

### Cell Culture and Treatment

All of the cell lines were purchased from the cell bank of the Chinese Academy of Sciences (Shanghai Institute of Cell Biology, Chinese Academy of Sciences, China) and were maintained under a humidified atmosphere of 5% CO_2_ at 37 °C. Mouse immortalized hepatocytes AML12 were cultured in DMEM/Ham's F12 supplemented with 10% fetal bovine serum (FBS), and the mouse spermatocyte‐derived GC‐2spd(ts) cell line was grown in DMEM with 10% FBS. The 3T3‐L1 preadipocytes were cultured in DMEM supplemented with 10% bovine calf serum. For adipogenic induction, the culture medium was replaced with DMEM containing 10% FBS, 1 µm dexamethasone (Sigma, USA), and 0.5 mm 3‐isobutyl‐1‐methylxanthine (IBMX, Sigma, USA) once the cells achieved 70–80% confluence, and then incubated for 48 h.

Subsequently, the cells were treated with DMEM containing 10% FBS plus 10 µg mL^−1^ insulin for an additional 48 h, followed by a transition to DMEM containing 10% FBS for 6–8 days.

For inhibition or overexpression of miRNAs, AML12 cells were transfected with 10 nm miR149‐5p mimic, miR150‐5p mimic, miR700‐5p mimic or mixture‐mimics (Ribo Bio, China) for 48 h (Figure S8A–D, Supporting Information). For the overexpression of Lepr, AML12 cells were transfected with pEGFP‐Lepr (Sangon Biotech, China) using Lipofectamine 3000 reagent (Life Technologies, USA) (Figure S9A, Supporting Information). For siRNA experiments, GC‐2spd cells were transfected with 50 nm siRNA oligonucleotides specific against Pparg (Hanbio, China) and Srebf1 (Santa Cruz, USA, sc‐36558), Fto (SBO Medical Biotechnology, Shanghai, China) for 48 h (Figure 2L,N).

### Sperm Isolation

Mature sperm was collected from the caudal epididymis of F0 male mice at the end of treatment and were incubated in PBS at 37 °C for 30–60 min to allow sperm to diffuse into the medium. For Sperm count measurement, 10 µL of the sperm mixture was dropped on a glass slide and the count was quantified in a hemocytometer under a light microscope. The tissue debris was then removed by centrifugation at 400× *g* for 2 min. The harvested sperm were incubated with somatic cell lysis buffer for 30 min on ice, followed by centrifugation for 5 min at 4000× *g* and 4 °C. Afterward, sperm was washed twice with PBS and precipitated again at 12 000× *g* for 5 min. The sperm was finally resuspended in 1 mL TRIzol reagent (Thermo Fisher Scientific) or stored at −80 °C before being used for sequencing.

### miRNA and mRNA Microarray Analysis

For small RNA‐seq, total RNAs from sperm in F0 male mice were extracted using Trizol reagent. The quantity of RNA was determined and shown in Figure 2A,D. About 400 ng total RNAs per sample were used for small RNA library construction and amplicon sequencing (BGI, China). In addition, the liver tissues of F1 male mice were subjected to total RNA extraction, followed by transcriptome RNA sequencing using the Novogenes Hiseq 4000 platform (China). After quality control, high‐quality clean reads were obtained for downstream bioinformatics analyses. The DEGseq software was used to analyze differentially expressed miRNAs, whereby miRNAs exhibiting a Log2 (fold change) greater than 2 and a *q*‐value less than 0.05 were considered statistically significant. In the liver of male offspring, differentially expressed genes (DEGs) were identified using *DESeq2* with a Log2 fold change greater than 2 and adjusted *p*‐value (P_adj_) less than 0.05 as the parameters. All miRNAs or mRNAs identified as differentially expressed were further used for functional enrichment analysis. For miRNAs, pathway analysis was performed on the predicted mRNA targets based on miRWalk 2.0 (http://mirwalk.umm.uni‐heidelberg.de). KEGG pathway analysis and GO analysis were performed using the clusterProfiler R package (v4.10.0). The PPI networks were also constructed and visualized using the STRING website (https://string‐db.org/) and Cytoscape (version 3.9.1) software. Another bioinformatic analysis was performed using the OmicStudio tools (https://www.omicstudio.cn/tool).

### Serum Biochemical and Cytokine Measurement

After a 16 h fasting, serum TG, TC, AST, and ALT were determined using automatic biochemical analyzer (BS‐240VET, Mindray, China). Serum FFA and the extracted hepatic TC and TG were measured using enzymatic kits (Applygen Technologies, China). Serum levels of insulin (ExCell Bio, China), leptin (Sigma‐Aldrich, Merck Millipore, Germany), Lepr (Abcam, ab267584 UK) as well as cytokines IL6, Tnfα, and Il1β (ExCell Bio,) were measured by commercially available ELISA kits.

### The Metabolic Phenotype

For the intraperitoneal glucose tolerance test (IPGTT), F0 male mice and 48‐week‐old F1 offspring were fasted for 16 h and received intraperitoneal injection of 20% glucose at a dose of 2.0 g kg^−1^ body weight. For the insulin tolerance test (IPITT), 6‐h‐fasted mice were intraperitoneally injected with insulin (Novolin R, Denmark) at a dose of 0.75 U kg^−1^ body weight. Blood glucose levels were measured before and 15, 30, 60, and 120 min after glucose or insulin injection using a handheld glucometer (AccuChek Active; Roche, Germany).

At the age of 48 weeks, four F1 mice per group were randomly selected and individually placed in metabolic cages for 7 consecutive days to conduct measurements of whole‐body metabolic rates (Sable Systems International, USA). The initial 24 h data were not analyzed but considered as an adaptation phase. The parameters examined included energy expenditure (evaluated by indirect calorimetry), respiratory quotient (volume of oxygen consumed and volume of carbon dioxide produced, mL/kg/h), water and food consumption, and body mass. The data were collected for each mouse at 10 min intervals throughout the day. The calorimetry data and the energy expenditure data were analyzed using CalR, a web‐based analysis tool (https://calrapp.org/).^[^ ^73^ ^]^ CalR will examine the data for significant interaction effects between mass and mass‐dependent metabolic variables. If an interaction is detected for any metabolic variable, CalR will employ the generalized linear models to elucidate the exposure effect while controlling for mass influences. But if the interaction effect is absent, the model is rerun and the analysis of covariance results are displayed.

### Histological Analyses

For morphological analysis, the liver sections were stained with H&E. To assess hepatic steatosis, fixed liver tissues were embedded in optimum cutting temperature compound, cryosectioned, and subsequently stained with oil‐red‐O. Immunofluorescence staining for F4/80 was performed to evaluate liver inflammation. Quantitative analyses were performed with ImageJ software (National Institutes of Health, USA).

For transmission electron microscopy, fresh liver tissues were fixed with 2.5% glutaraldehyde, stained with 1% osmium tetroxide and uranyl acetate, dehydrated in a series of ethanol dilution, and embedded in an Araldite‐Epon mixture. The ultrathin sections were cut, mounted on copper grids, stained with uranyl acetate and lead citrate, and finally imaged by transmission electron microscope (HT‐7800, Hitachi, Japan). The mitochondria–ER proximity was quantified by measuring the fraction of mitochondrial membrane in contact with ER within a 50 nm range and normalized to the mitochondrial perimeter. Images were analyzed using the ImageJ software (NIH).

### Cell Viability with CCK8 Assays

The GC‐2spd cells were exposed to BPA for 24, 48, and 72 h. After stimulation, CCK8 solution (10%) was added to each well and incubated for 2 h at 37 °C. Then the absorbance was read on a spectrophotometer at a wavelength of 450 nm with a reference wavelength of 600 nm.

### Analysis of Intracellular Lipids

Intracellular TG contents were measured using a commercial kit (Applygen Technologies). Oil red O (Solarbio, China) and Bodipy FL C16 (Thermo Fisher Scientific) staining were employed to visualize neutral lipid accumulation. Imaging was conducted using a Nikon Eclipse Ts2 inverted microscope (Nikon, Japan) and an LSM 880 Zeiss confocal microscope (Zeiss, Germany) equipped with a 63× oil immersion objective, respectively. The signal intensity of the lipid droplets was quantified by ImageJ software (NIH).

### Determination of ATP Levels

The GC‐2spd cells were treated with BPA for 48 h and the intracellular ATP content was measured using the ATP Bioluminescence Assay Kit (Beyotime, China) following the manufacturer's instructions. ATP concentration was calculated by plotting an ATP standard curve and was normalized to the total protein concentration of the cell lysate.

### Total m6A RNA Methylation Level

Quantification of m6A RNA methylation in the GC‐2spd cells was detected using m6A RNA methylation quantification kit (Abcam) following the manufacturer's protocol. Briefly, 300 ng RNA was incubated with capture antibody for 60 min. After that, the detection antibody and enhancer solution were added. The wells were subsequently subjected to a washing procedure, followed by incubation with a substrate to induce color development. The resulting absorbance was monitored at a wavelength of 450 nm using a microplate reader (Tecan, Infinite E Plex, Switzerland).

### mRNA Stability Assay

The GC‐2spd cells were treated with 20 µm BPA for 48 h, followed by the addition of transcription inhibitor actinomycin D (5 µg mL^−1^, Sigma) for 0, 3, and 6 h. Cells were then harvested for RNA extraction. The mRNA expression for *Srebf1* at the indicated time was calculated and normalized by β‐actin.

### Chromatin Immunoprecipitation (ChIP)

Two *Pparg*‐binding sites in the *Dicer1* promoter region were identified by the ChIP (Millipore, USA). In brief, GC‐2spd cells were cross‐linked with 1% formaldehyde and lysed with SDS. The lysates were sonicated to generate 300–600 bp DNA fragments. Then the sonicated samples were diluted in ChIP dilution buffer and precleared with salmon sperm DNA/protein A agarose at 4 °C for 30 min with agitation. 1% of each sample was saved for the input control. Soluble chromatin complexes were immunoprecipitated using anti‐Pparg (Cell Signaling Technology, USA), anti‐RNA Pol II (Abcam, ab5095), or IgG (Cell Signaling Technology) with constant rotation at 4 °C overnight. The protein–DNA–antibody complex was pulled down by protein A agarose–salmon sperm DNA beads, followed by low‐salt, high‐salt, LiCl, and TE buffer sequential washes. The eluted complex and input samples were reverse‐crosslinked using 5 m NaCl at 65 °C overnight. After digestion with proteinase K and RNase, ChIP DNA was extracted using phenol/chloroform method. qPCR was performed on the immunoprecipitated DNA and input DNA using primers designed to cover putative Pparg binding sites within the promoter region of Dicer1 (Table S3, Supporting Information). The enrichment of the targeted region of DNA was evaluated by % input and was represented as fold enrichment compared to IgG control.

### Luciferase Reporter Assay

Targeting of *Lepr* 3′UTR by miR149‐5p, miR150‐5p, and miR700‐5p was validated by the luciferase reporter assay. DNA sequences encompassing the target 9‐base pair region (905‐913: TGGGAGCCA) within the 3′ UTR of Lepr, along with its mutants (TATCGAATA), were synthesized (Sangon Biotech) and subsequently cloned into the pmirGLO Dual‐Luciferase miRNA Target Expression Vector (Promega, USA). AML12 cells were cotransfected with the indicated luciferase reporter vectors and miRNA mimic (single miR149‐5p mimic, miR150‐5p mimic, miR700‐5p mimic, or mixture‐mimics) for 48 h. The relative luciferase activities were measured using a dual luciferase reporter assay kit (Promega).

### SA‐β‐Galactosidase Staining

To assess the presence of senescent cells, AML12 cells were fixed and incubated with β‐galactosidase staining solution (pH 6.0, Beyotime) overnight at 37 °C. Imaging was performed on Nikon Eclipse Ts2 inverted microscope. SA‐β‐gal‐positive cells were quantified and analyzed by ImageJ software.

### Ethynyl Deoxyuridine (EdU) Cell Proliferation Staining

Cell proliferation was determined by EdU incorporation assay (Ribo Bio). Following treatment, AML12 cells were incubated with EdU at 37 °C for 2 h. Subsequently, the cells were fixed with 4% paraformaldehyde for 30 min, neutralized with 2 mg mL^−1^ glycine for 5 min, permeated with 0.5% tritonX‐100 for 10 min, and stained with Apollo fluorescent dyes for 30 min in the dark. After that, cell nuclei were stained blue using Hoechst 33342, and EdU‐positive cells were visualized and quantified using a Nikon Eclipse Ts2 inverted microscope.

### RNA Extraction and Quantitative Real‐Time PCR Assay

Total RNA was extracted using TRIzol Reagent (Sangon Biotech) and cDNA was obtained using PrimeScript RT Master Mix (Takara Biotechnology, Japan) or ReverTra Ace qPCR RT Master Mix (Toyobo, Japan). Real‐time PCR assays were performed using KOD SYBR qPCR Mix (Toyobo) on the Roche LightCycler 96 machine (Roche, Switzerland). The primers for mRNA, miRNAs, pri‐miRNAs, and pre‐miRNAs were listed in Tables S2, S4, and S5 of the Supporting Information.

### Western Blot Analysis

Total protein was prepared using RIPA (Thermo Fisher Scientific). Proteins from both cell and tissue lysates were subsequently separated via SDS‐PAGE and transferred to polyvinylidene difluoride membrane (PALL, USA). The membrane was then incubated with a specific primary antibody overnight, followed by incubation with HRP‐conjugated secondary antibody. Antibody‐reactive bands were visualized using ECL chemiluminescence detection system (WesternBright ECL Western Blotting HRP Substrate, USA). The intensity of the bands was quantified using ImageJ software. Detailed information on the primary antibodies and dilutions was provided in Table S6 of the Supporting Information.

### Statistical Analysis

All data were presented as the mean ± SD. The number of samples and experimental repeats were indicated in the figures and corresponding legends. Statistical analysis was performed using the Graph Pad Prism 7. The comparisons between the two groups were performed by a two‐tailed Student's *t*‐test. And one‐way analysis of variance followed by the Bonferroni post hoc test was used to determine statistical significance among multiple groups. Statistical significance values were set at *p* < 0.05, *p* < 0.01, *p* < 0.001, and *p* < 0.0001.

## Conflict of Interest

The authors declare no conflict of interest.

## Supporting information



Supporting Information

## Data Availability

The data that support the findings of this study are available from the corresponding author upon reasonable request.
