# Supplementary material for: Multi‐miRNAs‐Mediated Hepatic Lepr Axis Suppression: A Pparg–Dicer1 Pathway‐Driven Mechanism in Spermatogenesis for the Intergenerational Transmission of Paternal Metabolic Syndrome
Source: Adv Sci (Weinh). 2025 Jan 10;12(9):2410831. doi: 10.1002/advs.202410831 (PMC11884570; doi:10.1002/advs.202410831)
Supplement: Supplementary file 1 — Supporting Information [file ADVS-12-2410831-s001.docx]

**Multi-miRNAs-Mediated Hepatic Lepr Axis Suppression: A Pparg-Dicer1 Pathway-Driven Mechanism in spermatogenesis for the Intergenerational Transmission of Paternal Metabolic Syndrome**

Yi Lin^1^^,3,#,*^, Xiuye Ni^1^^, #^, Lin Zhu^1, #^, Yilong Lin^2, #^,Cai Peng^2^, Zhao Lei^1^, Yihui Wang^1^, Huan Wang^1^, Xiang You^2^, Juan Li^2^, Heqing Shen^1,*^, Jie Wei^2,*^

^1^State Key Laboratory of Vaccines for Infectious Diseases, Xiang An Biomedicine Laboratory & State Key Laboratory of Molecular Vaccinology and Molecular Diagnostics, School of Public Health, Xiamen University.

^2^Department of Basic Medical Sciences, School of Medicine, Xiamen University, Xiamen 361102, China.

^3^Fujian Key Laboratory of Coastal Pollution Prevention and Control, Xiamen University

**Supplementary Materials**

**Supplementary Figures**

**Figure S1**

**
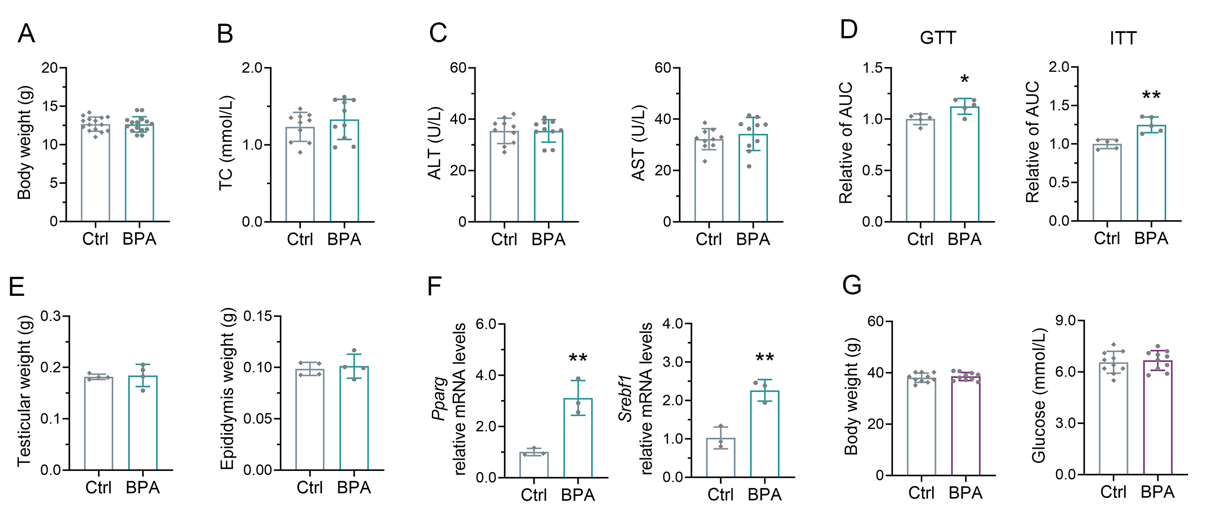
**

**Figure S1** **Maternal and paternal physiological parameters. A.**The body weight of F0 male mice before BPA exposure. **B.** Serum total cholesterol (TC) in F0 male mice. **C.** The serum concentrations of liver function indicators ALT and AST in F0 male mice. **D.** Quantification of the area under the blood glucose-time curve (AUC) during GTT and ITT. The curves were in Figure 1E. **E.** The weight of the testis and epididymis in F0 male mice. **F.** The mRNA expression of *Pparg* and *Srebf1* in the sperm of F0 male mice. *Gapdh* was used as a reference gene. **G.** The body weight and fasting blood glucose in F0 female mice. n = 3-10 mice/group as indicated. Data were presented as the mean ± SD. **p*<0.05, ***p*<0.01, *vs.* corresponding controls.

**Figure S2**

**
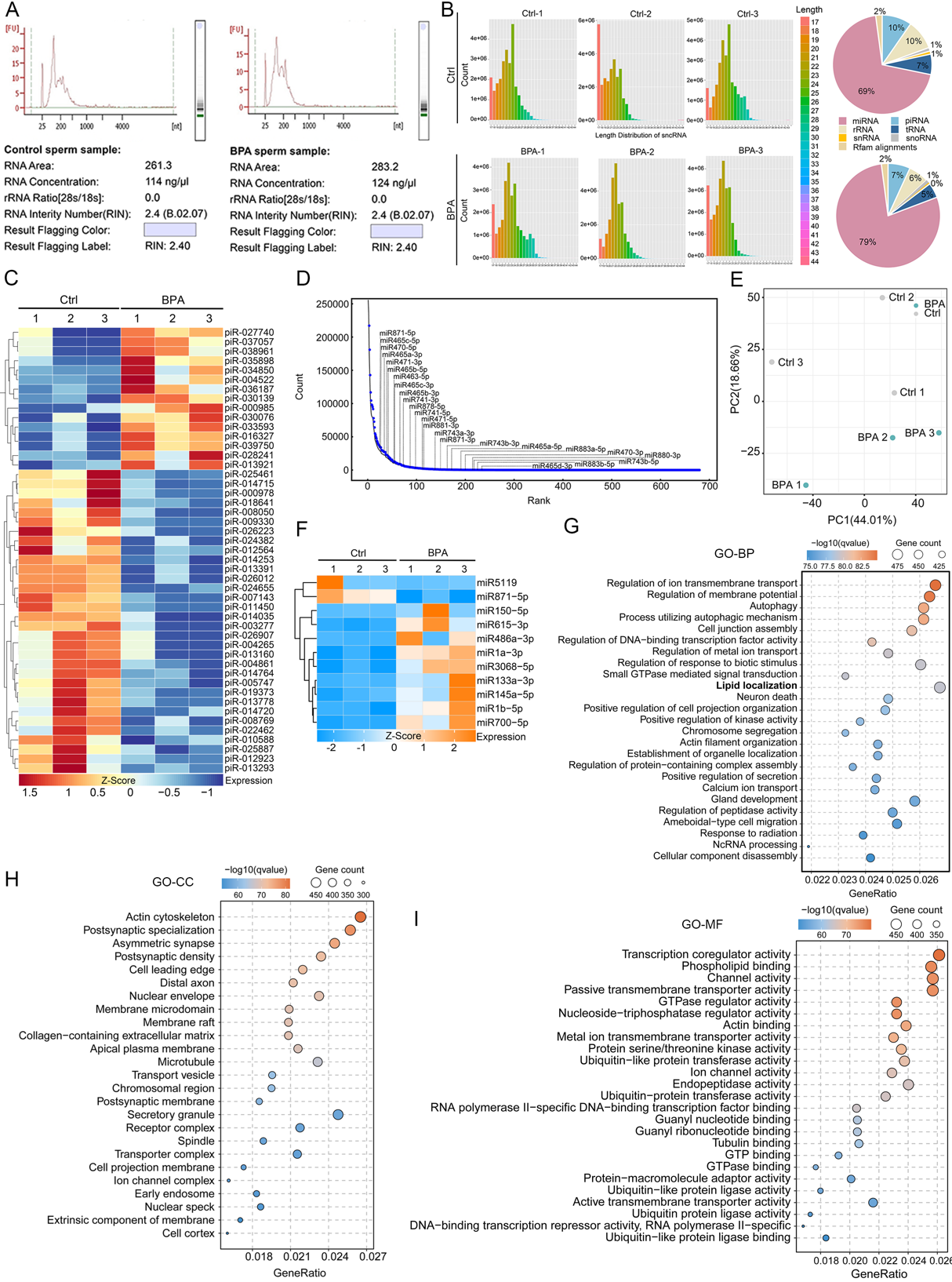
**

**Figure S2 The small non-coding RNA (sncRNAs)-sequencing data of sperm obtained from F0 male mice. A.** Electrophoretic size distribution of RNAs extracted from mature sperm of F0 male mice. The extracted sperm RNA was of high quality and devoid of somatic RNA contamination. **B.** The size distribution of sequencing reads for sperm sncRNAs. The pie chart showed the abundance distribution of different sncRNA species in sperm from F0 mice. miRNA: microRNA; piRNA: piwi-interacting RNA; rRNA: ribosomal RNA; tRNA: transfer RNA; snRNA: small nuclear RNA; snoRNA: small nucleolar RNA; Rfam other sncRNA: other small non-coding RNAs  in Rfam database. **C.** The piRNAs with a differential expression of at least four-fold were identified and clustered for heatmap visualization. **D.** Read counts of X-linked miRNAs, mainly found in mammalian sperm and known as spermatogenesis-related miRNAs (spermiRs). **E.** The principal component analysis (PCA) used to examine the variance in sperm miRNA distribution between control and BPA-exposed F0 male mice. **F.** The clustered heatmap illustrating the differential expression of miRNAs, employing a four-fold threshold. **G-I.** The top 25 pathways of gene ontology (GO) enrichment analysis of differential miRNA target genes. Three GO categories, biological process (GO-BP, G), cellular component (GO-CC, H), and molecular function (GO-MF, I) were tested. For G, the glucose and lipid metabolism-related GO-BP pathways in Figure 1N were bolded. Color bars, the log (q value) of enrichment; Gene count, count of differential miRNA target genes in the indicated pathway; GeneRatio, differential gene count in the indicated pathway versus total differential gene count.

**Figure S3**


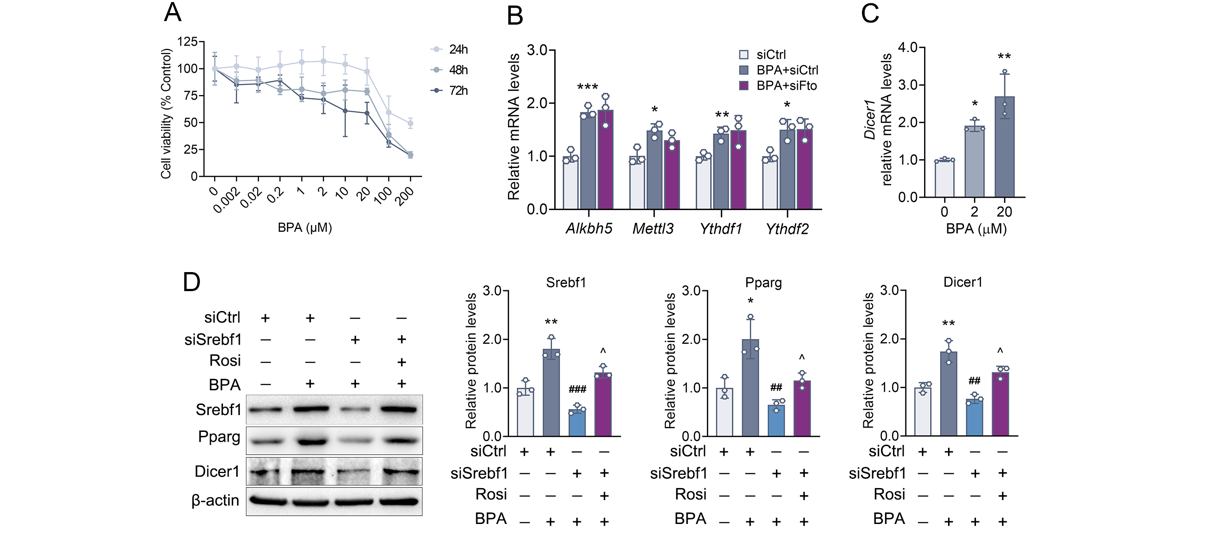


**Figure S3 The** **role of BPA exposure on the Srebf1-Pparg-Dicer1 signal pathway in** **spermatocytes.** **A.** The Viability of GC-2spd cells exposed to a concentration series of BPA (0.002-200 µM) for 24, 48, and 72 hours. **B.** The role of Fto inhibition on the mRNA expression of some BPA-upregulated m^6^A RNA methylation regulators. The *β-actin* was used as a reference gene for RT-PCR. The GC-2spd cells were co-treated with 20 µM BPA and 50 nM siFto for 48 h. **C.** The mRNA expression of the *Dicer1*. The *β-actin* was used as a reference gene. The GC-2spd cells were treated with 0, 2, and 20 µM BPA for 48 h. **D.** The protein expression of the Srebf1-Pparg-Dicer1 signaling pathway in spermatocytes. Data were normalized to β-actin. The GC-2spd cells were treated with 20 µM BPA, and then co-transfected with 50 nM siSrebf1, or further co-treated with 10 µM Rosiglitazone (a full agonist for Pparg) for 48 h as indicated. n=3 independent experiments. Data were presented as the mean ± SD. **p*<0.05, ***p*<0.01, ****p*<0.001, BPA and control siRNA (siCtrl) co-treated cells *vs.* siCtrl-treated cells. ##*p*<0.01, ###*p*<0.001, BPA and siFto (or siSrebf1) co-treated cells *vs.* BPA and siCtrl co-treated cells. ∧*p*<0.05, BPA, siSrebf1, and rosiglitazone co-treated cells *vs.* BPA and siSrebf1 co-treated cells.

**Figure S4**

**
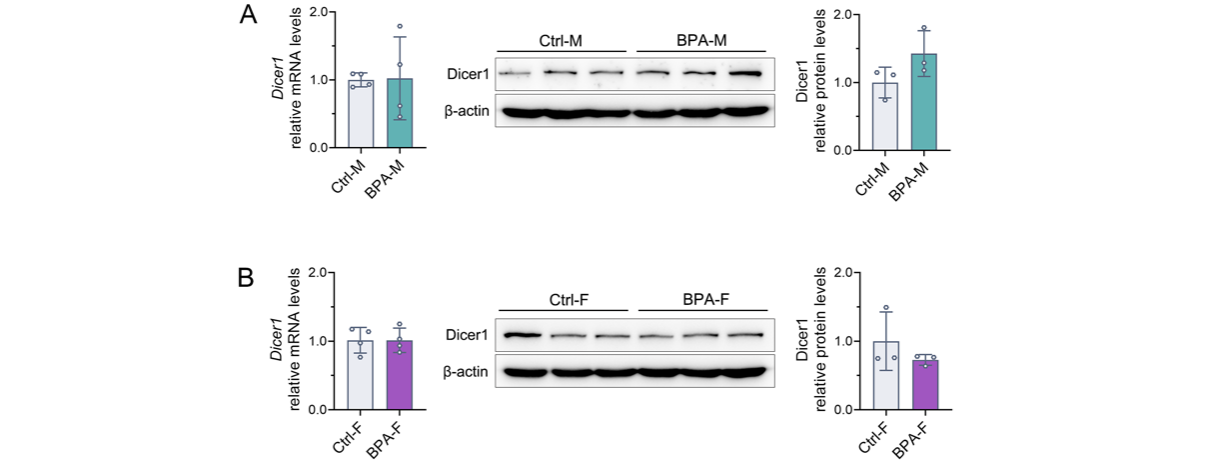
**

**Figure S4** The mRNA and protein expression of Dicer1 in the liver of F1 offspring. β-actin was used as an internal reference. “M” meant male offspring and “F” meant female offspring. n=3-4 mice/group as indicated. Data were presented as the mean ± SD.

**Figure S5**


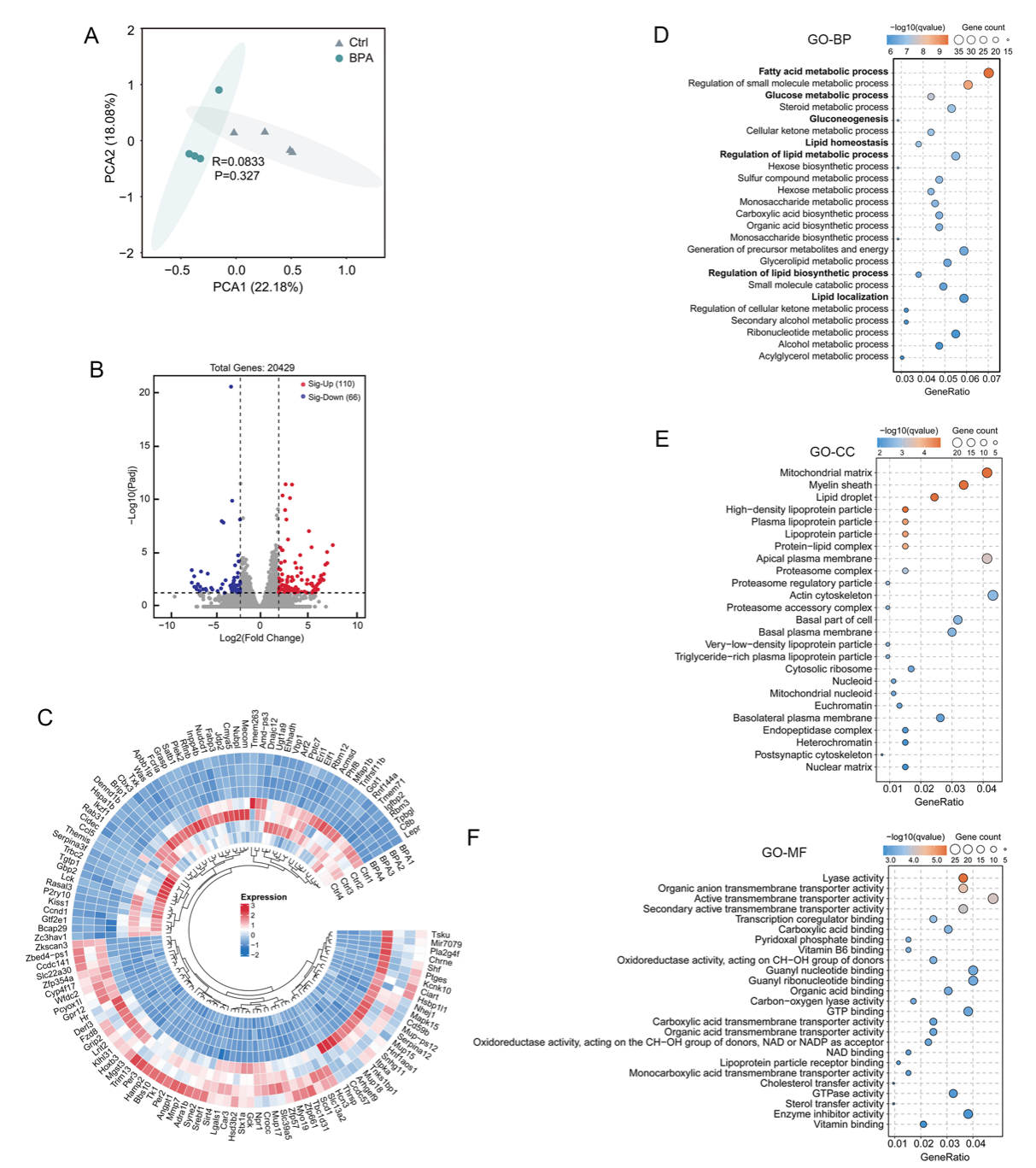


**Figure S5 Transcriptomic analyses of liver samples from F1 male offspring.** **A.** Principal component analysis (PCA). **B.** Volcanic map of differential expressed genes (DEGs) in the liver between paternal BPA-exposed male offspring and controls. The log 2-fold change (Ctrl/BPA) were plotted against the -log 10 adjusted *p*-values (Padj) for all genes. **C.** The cluster heat map of DEGs. Red and blue colors represented up-regulated and down-regulated genes, respectively. **D-F**. GO enrichment analysis of DEGs. The bubble chart displayed the top 25 biological processes (BP, D), cellular components (CC, E), and molecular functions (MF, F). For D, the glucose and lipid metabolism-related GO-BP pathways in Figure 4B were bolded. The color bars are the log (q value) of enrichment; Gene count is the count of differential genes in the indicated pathway; and GeneRatio is the differential gene count in the indicated pathway versus the total differential gene count. n=4 mice/group as indicated.

**Figure S6**

**
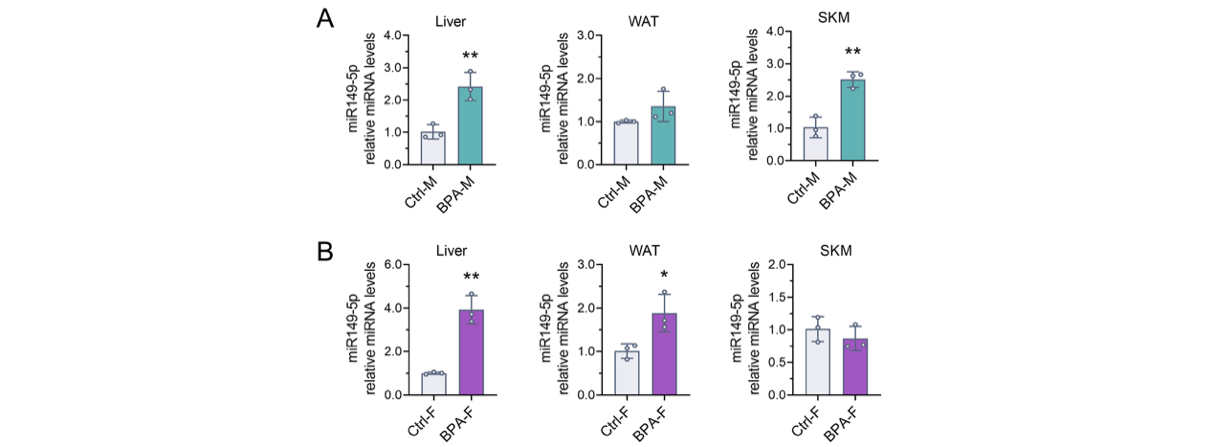
**

**Figure S6** Validation of miR149-5p expression in the metabolic organs, including liver, skeletal muscle, and adipose tissue in offspring. U6 was used as a loading control. n=6 mice/group as indicated. Data were presented as the mean ± SD. *p<0.05, **p<0.01, vs. corresponding controls.

**Figure S7**

**
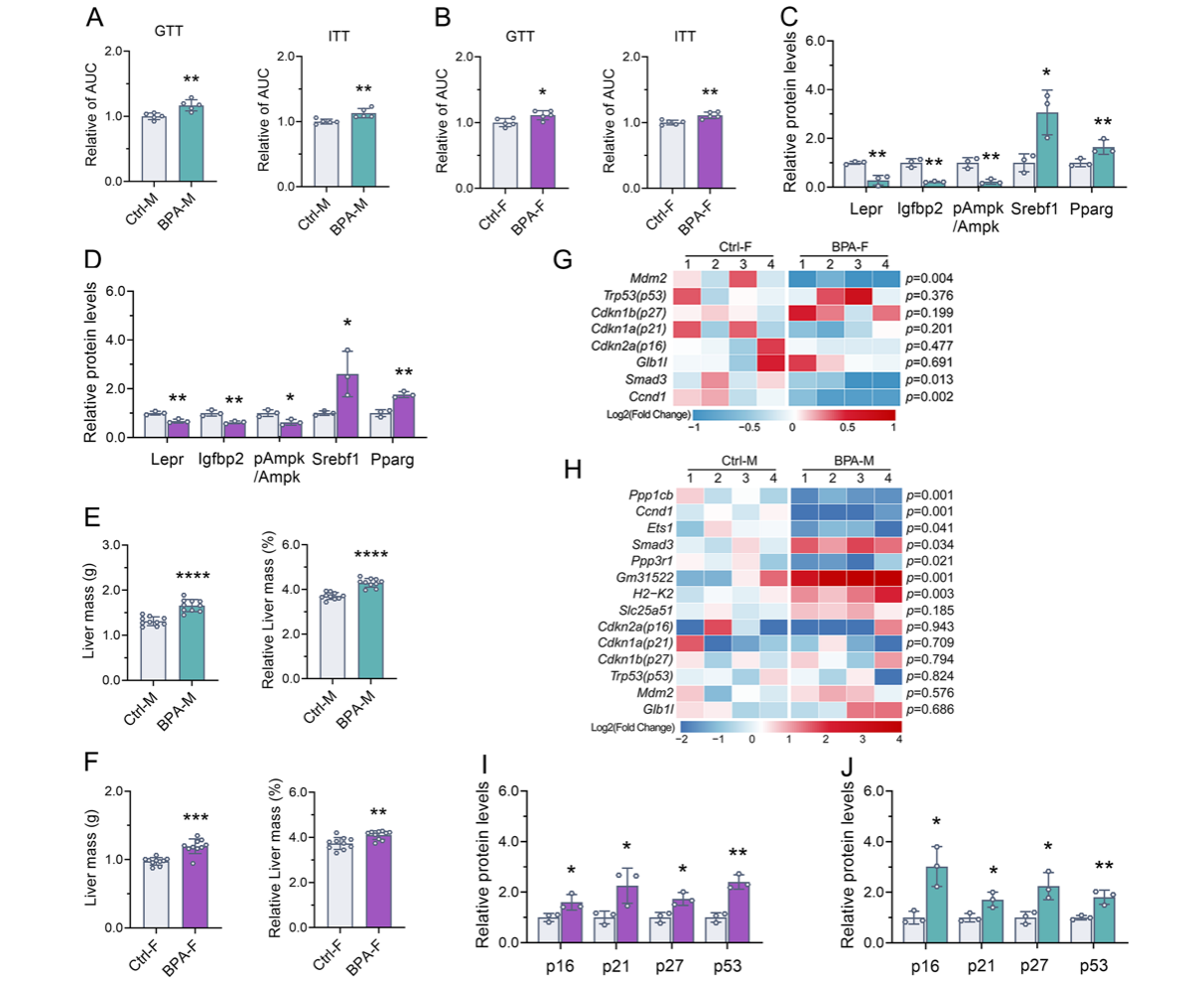
**

**Figure S7** **The role of paternal BPA exposure on the metabolic changes in the mice offspring.** “M” meant male offspring and “F” meant female offspring. **A-B.** The quantification of the area under the blood glucose-time curve (AUC) during GTT and ITT. The curves were in Figures 3E and F. **C-D.** Quantification of the protein expression of genes regulated by Lepr in the liver of male (C) and female offspring (D) in Figures 5L and N. β-actin was used as an internal reference. **E-F.** The liver weights and relative liver weights (% body weight). **G-H.** Heatmaps showing the relative expression of senescence-related gene expression in the liver of male and female offspring. For the male offspring (G), data represented were derived from the transcriptome sequencing, and for the females (H), data were obtained by RT-qPCR analysis, and *β-actin* was used as a reference gene. **I-J.** Quantification of the protein expression of several cellular senescence markers in the liver of male (I) and female offspring (J) in Figures 6H and J. β-actin was used as an internal reference. n=3-10 mice/group as indicated. Data were presented as the mean ± SD. **p*<0.05, ***p*<0.01, ****p*<0.001, *****p*<0.0001, *vs.* corresponding controls.

**Figure S8**


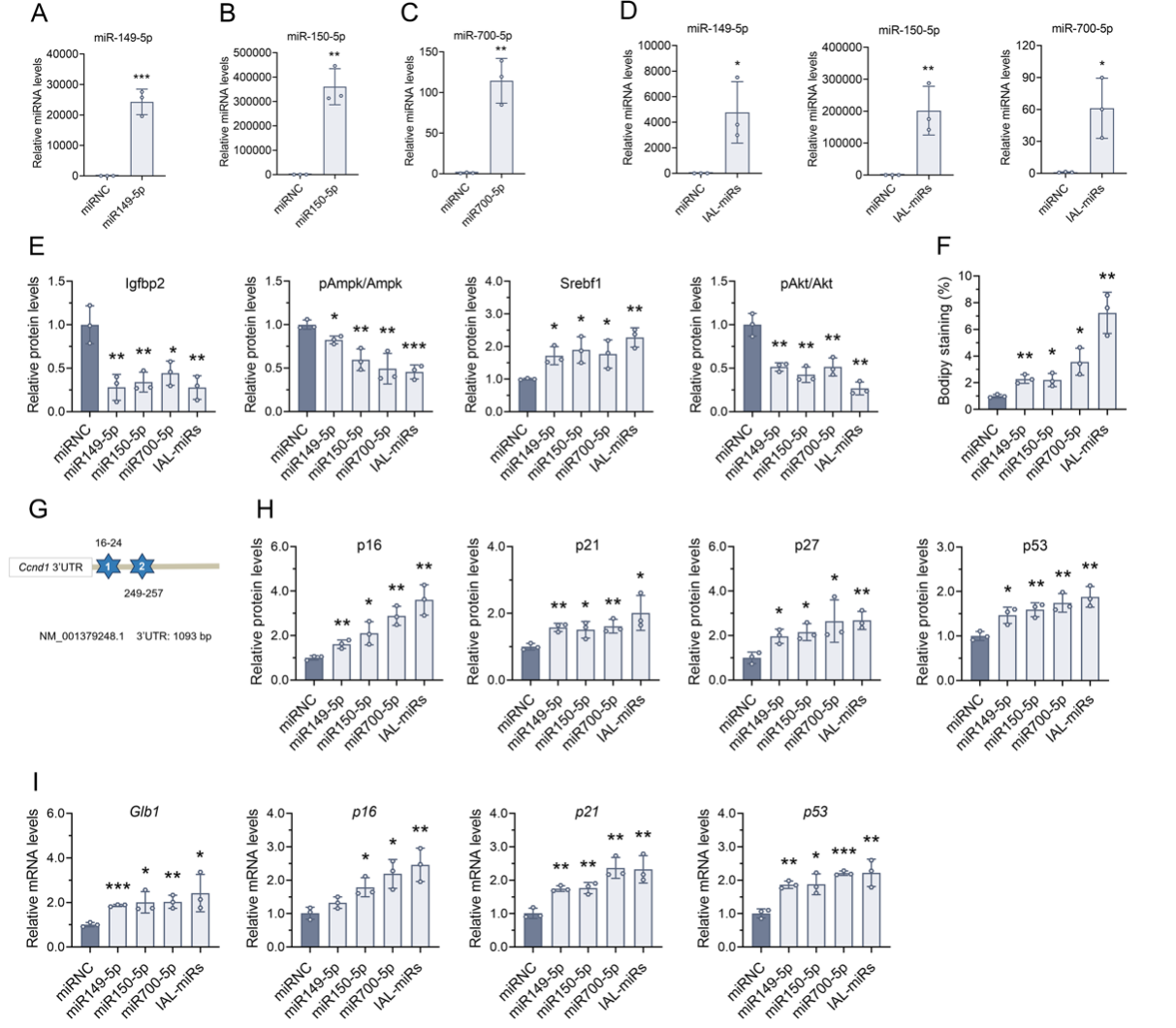


**Figure S8 The function of** **IAL-miRs in regulating metabolic homeostasis and senescence in AML12 cells.** AML12 cells were transfected with the single miR149-5p mimic, miR150-5p mimic, miR700-5p mimic, mixture-mimics (IAL-miRs), or control mimic (miRNC) for 48 h. **A-D.** The expression of miR149-5p, miR150-5p, and miR700-5p in AML12 cells transfected with the single mimic and mixture-mimics. U6 was used as a loading control. **E.** Quantification of the protein expression of downstream effector involved in Lepr-mediated regulation of glucose and lipid metabolism in Figure 5C. β-actin was used as an internal reference. **F**.Quantification of the total fluorescence of BODIPY-positive staining in Figure 5D. **G.** The diagram showed the “SMITE” sites in the 3′UTR of Ccnd1. **H.** Quantification of the protein expression of cellular senescence markers in Figure 6L. β-actin was used as an internal reference. I**.** The mRNA expression of several senescence-related genes. *β-actin* served as a reference gene. n=3 independent experiments/group. Data were presented as the mean ± SD. **p*<0.05, ***p*<0.01, ****p*<0.001, *vs.* corresponding controls.

**Figure S9**


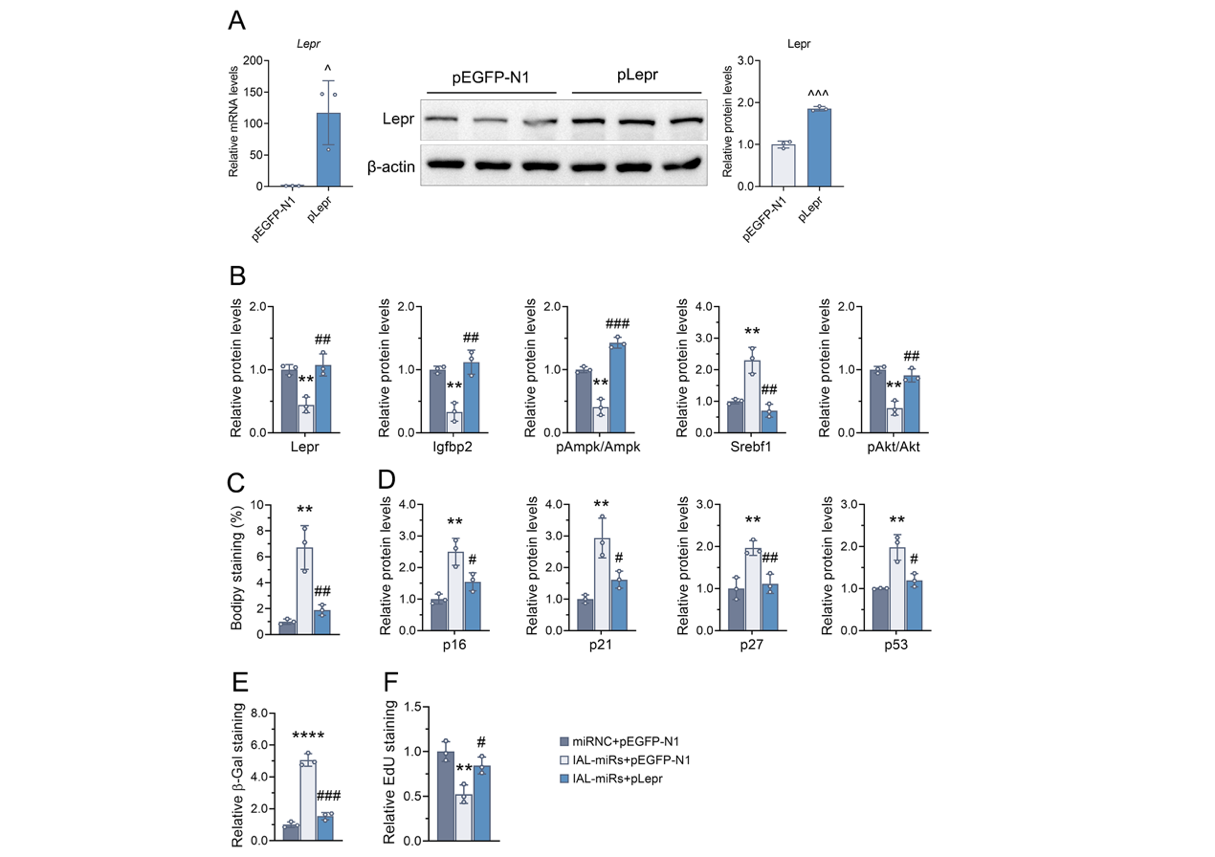


**Figure S****9** **Overexpression of Lepr protected the AML12 cells against the IAL-miRs-impaired lipid homeostasis and insulin signaling. A.** The mRNA and protein expression of Lepr in AML12 cells transfected with the pEGFP-N1 or pEGFP-Lepr (pLepr) for 48h. β-actin was used as an internal reference. **B-F** AML12 cells were co-transfected with the IAL-miRs mimics, pLepr, or corresponding controls for 48 h. **B.** Quantification of the protein expression of downstream effector involved in Lepr-mediated regulation of glucose and lipid metabolism in Figure 5F. β-actin was used as an internal reference. **C**. Quantification of the total fluorescence of BODIPY-positive staining in Figure 5G. **D.** Quantification of the protein expression of cellular senescence markers in Figure 6P. β-actin was used as an internal reference. **E.** Quantification of the senescence-associated SA-β-gal staining in Figure 6Q. **F.** Quantification of the EdU-labeled cells in Figure 6Q. n=3 independent experiments/group. Data were presented as the mean ± SD. ∧*p*<0.05, ∧∧∧*p*<0.001, AML12 cells transfected with pEGFP-N1 vs. AML12 cells transfected pLepr. ***p*<0.01, ****p*<0.001, AML12 cells co-transfected with control mimic and pEGFP-N1 vs. AML12 cells co-transfected IAL-miRs mimics and pEGFP-N1. #*p*<0.05, ##*p*<0.01, ###*p*<0.001, AML12 cells co-transfected IAL-miRs mimics and pEGFP-N1 vs. AML12 cells co-transfected IAL-miRs mimics and pLepr.

**Figure S10**

**
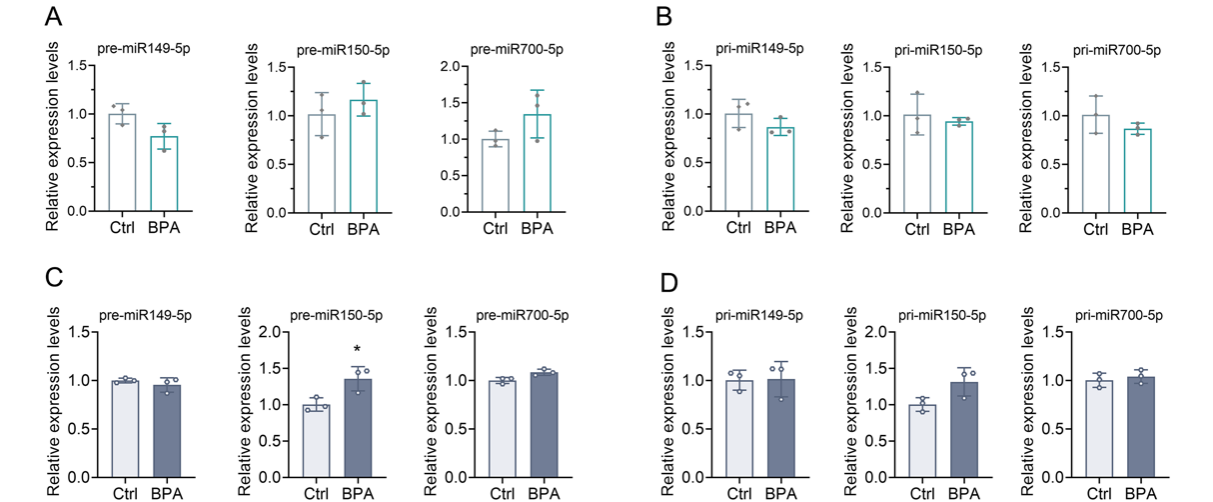
**

**Figure S10** **The role of BPA exposure on the expression of pri-IAL-miRs and pre- IAL-miRs in sperm of F0 male mice (A-B) and spermatocytes (C-D).** The GC-2spd cells were treated with 20 µM BPA for 48 h. Data were presented as the mean ± SD. For A-B, n=3 mice/ group, and for C-D, n=3 independent experiments/group. Data were presented as the mean ± SD.

**Supplementary Tables**

**Supplementary** **Table S1**

**Table S1** The Differentially expressed miRNAs in sperm from F0 male mice (Fold change ≥2, q<0.05).

| **miRNAs** | **Log2 fold change (BPA/Ctrl)** | **-Log10(q value)** |
| --- | --- | --- |
| miR23b-3p | 1.169673683 | 1.347889595 |
| miR23a-3p | 1.210195017 | 1.347889595 |
| miR100-5p | 1.212819892 | 1.347889595 |
| miR145a-3p | 1.349913922 | 1.305255896 |
| miR143-3p | 1.36809665 | 1.711747992 |
| miR26a-5p | 1.387260934 | 1.557095856 |
| miR574-5p | 1.576192682 | 1.552778596 |
| miR149-5p | 1.6132937 | 1.585769963 |
| miR181a-5p | 1.718145649 | 2.465544264 |
| miR29b-3p | 1.796740129 | 1.399559273 |
| miR214-3p | 1.890622698 | 1.487203694 |
| **miR145a-5p** | **2.003953996** | **1.347889595** |
| **miR3068-5p** | **2.132607973** | **2.736078442** |
| **miR615-3p** | **2.230859865** | **2.298961251** |
| **miR700-5p** | **2.236183451** | **1.904912142** |
| **miR1b-5p** | **2.268346017** | **2.143915352** |
| **miR1a-3p** | **2.28008595** | **5.246582469** |
| **miR486a-3p** | **2.307108971** | **1.347889595** |
| **miR133a-3p** | **2.704570918** | **3.698796279** |
| **miR150-5p** | **2.786079229** | **2.298961251** |
| **miR-5119** | **-3.923030157** | **1.347889595** |
| **miR-871-5p** | **-2.001759943** | **2.736078442** |
| miR-878-5p | -1.701972006 | 1.490030265 |
| miR-34b-3p | -1.614951542 | 1.436073759 |
| miR-463-5p | -1.599166437 | 2.143915352 |
| miR-128-3p | -1.585454643 | 2.143915352 |
| miR-499-5p | -1.532143444 | 2.014282852 |
| miR-743a-5p | -1.490704282 | 2.014282852 |
| miR-350-3p | -1.458178241 | 1.467315336 |
| miR-449a-5p | -1.320619266 | 1.50901135 |
| miR-470-5p | -1.259846384 | 1.425815287 |

The miRNAs in sperm that exhibited an upregulation exceeding four-fold following BPA exposure were highlighted in bold.

**Supplementary Table S2**

**Table S2** Primers used in qRT-PCR analyses of mRNAs.

| Gene | Primer sequence (from 5’→3’) |
| --- | --- |
| *Acaca* | Forward: AAGGCTATGTGAAGGATG  Reverse: CTGTCTGAAGAGGTTAGG |
| *Acacb* | Forward: CTTGCTTCTCTTTCTGACTTG  Reverse: GGCTTCCACCTTACTGTTG |
| *Ago2* | Forward: CTCACCTGGTGGCCTTCC  Reverse: AGAGGTATGGCTTCCTTCAGC |
| *Alkbh5* | Forward:TGTGCTCAGTGGGTATGCTG  Reverse:CTGACAGGCGATCTGAAGCA |
| *Apob* | Forward: TGTACAACTGGTCAGCCTCCTACAC  Reverse: TGGTGTAGAGATCCATCACAGGAC |
| *β-actin* | Forward: GCACCACACCTTCTACAATG  Reverse: GGGGTGTTGAAGGTCTCAAAC |
| *Ccnd1* | Forward: AGACCATTCCCTTGACTGC  Reverse: AAGCAGTTCCATTTGCAGC |
| *Cpt1a* | Forward:TGGCATCATCACTGGTGTGTT  Reverse: GTCTAGGGTCCGATTGATCTTTG |
| *Cd36* | Forward: AGATGACGTGGCAAAGAACAG  Reverse: CCTTGGCTAGATAACGAACTCTG |
| *Cdkn2a* | Forward: AATCTCCGCGAGGAAAGC  Reverse: GTCTGCAGCGGACTCCAT |
| *Cdkn1a* | Forward: TTGCACTCTGGTGTCTGAGC  Reverse: TCTGCGCTTGGAGTGATAGA |
| *Cdkn1b* | Forward: TTGGGTCTCAGGCAAACTCT  Reverse: TCTGTTCTGTTGGCCCTTTT |
| *Dgcr8* | Forward:GGGGTTCCTTACTACGCATGT  Reverse: CACACTCTTGTCAGAGGTCTCCT |
| *Dicer1* | Forward:AGATGGAGGCGGAGTTCAG  Reverse: CAATGAGCAGGTTGGTCTCA |
| *Dnmt1* | Forward:CTTCACCTAGTTCCGTGGCTA  Reverse: CCCTCTTCCGACTCTTCCTT |
| *Dnmt3a* | Forward:GCACCAGGGAAAGATCATGT  Reverse: CAATGGAGAGGTCATTGCAG |
| *Dnmt3b* | Forward:GGATGTTCGAGAATGTTGTGG  Reverse: GTGAGCAGCAGACACCTTGA |
| *Drosha* | Forward: ATGCAAGGCAATACGTGTCAT  Reverse: TTTTGGGGTCTGAAAGCTGGT |
| *Egfr* | Forward:CTCCACTGTCCAGCTCATTAC Reverse: CCAGGTAGTTCATGCCCTTT |
| *Fasn* | Forward: GCTGCGGAAACTTCAGGAAAT  Reverse: AGAGACGTGTCACTCCTGGACTT |
| *Fatp2* | Forward: TCCTCCAAGATGTGCGGTACT  Reverse: TAGGTGAGCGTCTCGTCTCG |
| *Fatp5* | Forward: CTACGCTGGCTGCATAAAGATG  Reverse: CCACAAAGGTCTCTGGAGGAT |
| *Fto* | Forward: GACACTTGGCTTCCTTACCTG  Reverse:CTCACCACGTCCCGAAACAA |
| *Gapdh* | Forward: CAAGGAGTAAGAAACCCTGGACC  Reverse: CGAGTTGGGATAGGGCCTCT |
| *Glb1* | Forward: TGGCTGGGCTGAATGCTATC  Reverse: TGGTTGGGGTTCATGGAAGTT |
| *Igfbp2* | Forward: CCTTGCCAGCAGGAGTTG  Reverse: TCCGTTCAGAGACATCTTGC |
| *Lepr* | Forward: ACCTTCTCCAACCTGAAAGC  Reverse: AATTCAGCATAGCGGTGATG |
| *Mdm2* | Forward: TGAGGTCTATCGGGTCACAG  Reverse: GGGAGGATTCATTTCATTGC |
| *Mettl3* | Forward:CTGGGCACTTGGATTTAAGGAA  Reverse: TGAGAGGTGTAGCAACTT |
| *Mettl14* | Forward:CTGAGAGTGCGGATAGCATTG  Reverse:GAGCAGATGTATCATAGGAAGCC |
| *Mttp* | Forward:CCGGTCAAGCGTTGCATTTC  Reverse:AGAGCATTTCGTCTGTTTCGC |
| *Ppara* | Forward: CTGGTCTTAACCGGCCCAAT  Reverse: TGCACATAGCCAGAAGGGTG |
| *Pparg* | Forward: CCCACCAACTTCGGAATCAG  Reverse: TGCTGGAGAAATCAACTGTGGTA |
| *Scd1* | Forward: TGCTATCGGGGTGTTAATGA  Reverse: TCTTGTGGCATGGTTAATCCTA |
| *Smad3* | Forward: GGATGGTCGGCTGCAGGTGTCC  Reverse: TGTTGAAGGCAAACTCACAGAGC |
| *Srebf1* | Forward: TTGTGGAGCTCAAAGACCTG  Reverse: TGCAAGAAGCGGATGTAGTC |
| *Trp53* | Forward: TGGAGGAGTCACAGTCGGATA  Reverse: GTCCATGCAGTGAGGTGATG |
| *Wtap* | Forward:GAACCTCTTCCTAAAAAGGTCCG  Reverse: TTAACTCATCCCGTGCCATAAC |
| *Xpo5* | Forward: TGATCCTGTTTGGAGATGTCG  Reverse: CACATAGCAGATTTCCCAGTG |
| *Ythdc1* | Forward:TTGGGCTTGAAGTTGATAGAGC  Reverse: TCTCAGTGTTGTTGCCCTGTT |
| *Ythdc2* | Forward:GGTCCGATCAATCATCTGT  Reverse: GAAGTAACGAATAGGCATGT |
| *Ythdf1* | Forward:TGGGAGTGGACATTTCTGTG  Reverse: TTCTAAGGGCACCTCCTGTG |
| *Ythdf2* | Forward: GAGCAGAGACCAAAAGGTCAAG  Reverse: CTGTGGGCTCAAGTAAGGTTC |
| *Ythdf3* | Forward:GAGCATGGTAATAAGCGTTTGG  Reverse: CAACAGACTTCATTTCAGCCAC |

*Acaca, acetyl-Coenzyme A carboxylase alpha; Acacb, acetyl-Coenzyme A carboxylase beta; Ago2, argonaute RISC catalytic subunit 2; Alkbh5, alkB homolog 5, RNA demethylase; Apob, apolipoprotein B;β-actin, actin, beta; Ccnd1, cyclin D1; Cpt1a, carnitine palmitoyltransferase 1A;Cd36, CD36 molecule; Cdkn2a, cyclin dependent kinase inhibitor 2A, P16; Cdkn1a cyclin dependent kinase inhibitor 1A, P21; Cdkn1b, cyclin dependent kinase inhibitor 1B, P27;* *Dgcr8,* *DGCR8 microprocessor complex subunit;* *Dicer1*,*dicer 1, ribonuclease type III; Dnmt1,DNA methyltransferase 1; Dnmt3a,* *DNA methyltransferase 3A; Dnmt3b, DNA methyltransferase 3B;* *Drosha, drosha, ribonuclease type III; Egfr, epidermal growth factor receptor, liver; Fasn, fatty acid synthase;Fatp2, fatty acid transporter member 2; Fatp5, fatty acid transporter member 5; Fto, FTO alpha-ketoglutarate dependent dioxygenase; Gapdh, glyceraldehyde-3-phosphate dehydrogenase; Glb1, galactosidase beta 1; Igfbp2, insulin like growth factor binding protein 2; Lepr, leptin receptor; Mdm2, MDM2 proto-oncogene; Mettl3, methyltransferase 3, N6-adenosine-methyltransferase complex catalytic subunit; Mettl14, methyltransferase 14, N6-adenosine-methyltransferase subunit; Mttp, microsomal triglycerid transfer protein; Ppara, peroxisome proliferator activated receptor alpha; Pparg, peroxisome proliferator activated receptor gamma; Scd1, stearoyl-Coenzyme A desaturase 1; Smad3,SMAD family member 3;Srebf1, sterol regulatory element binding transcription factor 1; Trp53, transformation related protein 53, P53; Wtap, WT1 associating protein; Xpo5, exportin 5; Ythdc1, YTH domain containing 1; Ythdc2, YTH domain containing 2; Ythdf1, YTH N6-methyladenosine RNA binding protein 1; Ythdf2, YTH N6-methyladenosine RNA binding protein 2; Ythdf3, YTH N6-methyladenosine RNA binding protein 3.*

**Supplementary Table S3**

**Table S****3** Sequences of primers used for ChIP in Figure 2O

| Target | Primer sequence (from 5’→3’) |
| --- | --- |
| ARE1 | Forward: AACAACCACTACGTGGGACTG  Reverse: GGTACTCCAGGTGAGGGATG |
| ARE2 | Forward: AAGTTCCAGGACAGCCAGAG  Reverse: CTCCTTGTCCATTAGCAGATGT |

**Supplementary Table S4**

Table S4 Primers used in qRT-PCR analyses of miRNA

| miRNA | Genbank or miRBase seq# | Primer sequence (from 5’→3’) |
| --- | --- | --- |
| miR1a-3p | MIMAT0000123 | Reverse transcription:  gtcgtatccagtgcgtgtcgtggagtcggcaattgcactggatacgactATACAT  Forward: ggggAACATTCAACGCTGTC  Reverse: ggggTGGAATGTAAAGAAGT |
| miR1b-5p | MIMAT0005835 | Reverse transcription:  gtcgtatccagtgcgtgtcgtggagtcggcaattgcactggatacgactTGGAAT  Forward:ggggTACATACTTCTTTACA  Reverse: TGCGTGTCGTGGAGTC |
| miR133a-3p | MIMAT0000145 | Reverse transcription:  tggagtcggcaattgcactggatacgactCAGCTG  Forward: ggggTTTGGCCCCTTCAAC  Reverse: TGCGTGTCGTGGAGTC |
| miR145a-5p | MIMAT0000157 | Reverse transcription:  gtcgtatccagtgcgtgtcgtggagtcggcaattgcactggatacgactAGGGAT  Forward: ggggGTCCAGTTTTCCCAGGA  Reverse: TGCGTGTCGTGGAGTC |
| miR149-5p | MIMAT0000159 | Reverse transcription:  gtcgtatccagtgcgtgtcgtggagtcggcaattgcactggatacgactGGGAGT  Forward: ggggTCTGGCTCCGTGTCTT  Reverse: TGCGTGTCGTGGAGTC |
| miR150-5p | MIMAT0000160 | Reverse transcription:  tggagtcggcaattgcactggatacgactCACTGG  Forward: ggggTCTCCCAACCCTTGTA  Reverse: TGCGTGTCGTGGAGTC |
| miR3068-5p | MIMAT0014842 | Reverse transcription:  gtcgtatccagtgcgtgtcgtggagtcggcaattgcactggatacgactGGTTAG  Forward: ggggTTGGAGTTCATGCAAG  Reverse: TGCGTGTCGTGGAGTC |
| miR486a-3p | MIMAT0017206 | Reverse transcription:  gtcgtatccagtgcgtgtcgtggagtcggcaattgcactggatacgactATCCTG  Forward: ggggCGGGGCAGCTCAGTAC  Reverse: TGCGTGTCGTGGAGTC |
| miR615-3p | MIMAT0003783 | Reverse transcription:  gtcgtatccagtgcgtgtcgtggagtcggcaattgcactggatacgactAAGAGG  Forward: ggggTCCGAGCCTGGGTCTC  Reverse: TGCGTGTCGTGGAGTC |
| miR700-5p | MIMAT0017256 | Reverse transcription:  gtcgtatccagtgcgtgtcgtggagtcggcaattgcactggatacgactGCAAGC  Forward: ggggTAAGGCTCCTTCCTGT  Reverse: TGCGTGTCGTGGAGTC |
| U6 RNA | NR_004394.1 | Reverse transcription:  CGCTTCACGAATTTGCGTGTCAT  Forward: GCTTCGGCAGCACATATACTAAAAT (LNA)  Reverse: CGCTTCACGAATTTGCGTGTCAT |

**Supplementary Table S5**

**Table S5** Primers used in qRT-PCR analyses of pri-IAL-miRs and pre-IAL-miRs

| Gene | Primer sequence (from 5’→3’) |
| --- | --- |
| Pri-miR149-5p | Forward: 5-CAATGCATGGGCTTCTGG-3  Reverse: 5-CAAACACGGGAGTGAAGACA -3 |
| Pri-miR150-5p | Forward: 5- CTGTGCCTCAGACCCTGGTA -3  Reverse: 5- GGAGGGAGAAGCTGGAGTTG -3 |
| Pri-miR700-5p | Forward: 5- GGGGAGAAATACGAACTGCAC -3  Reverse: 5- TCTTCCTCTTCAGTTTCGCTCT -3 |
| Pre-miR149-5p | Forward: 5-GGCTCTGGCTCCGTGTCT-3  Reverse: 5-CTCCCTCCCTCCTCGGAC-3 |
| Pre-miR150-5p | Forward: 5- CCTGTCTCCCAACCCTTGT -3  Reverse: 5- CCAGGCCTGTACCAGGGT -3 |
| Pre-miR700-5p | Forward: 5- GAGTAAGGCTCCTTCCTGTGC-3  Reverse: 5- CTGGGGGTGGACTCGGTT-3 |

**Supplementary Table S6**

**Table S6** Information of antibodies used in this study

| **Antibodies** | **Source** | **Identifier** | **Dilution** |
| --- | --- | --- | --- |
| Rabbit Anti-Argonaute 2 (Ago2) | Cell Signaling Technology | Cat. #2897 | WB,1:1000 |
| Rabbit Anti-Akt | Cell Signaling Technology | Cat. #9272 | WB,1:1000 |
| Mouse Anti-Ampk | Proteintech | Cat. 66536-1-ig | WB, 1:500 |
| Rabbit Anti-Dgcr8 | Cell Signaling Technology | Cat. #6914 | WB, 1:500 |
| Mouse Anti-Drosha | Santa Cruz | Cat. sc-393591 | WB, 1:500 |
| Mouse Anti-Dicer1 | Santa Cruz | Cat. sc-136979 | WB, 1:500 |
| Mouse Anti-Exportin5 | Santa Cruz | Cat. sc-271036 | WB, 1:100 |
| Rabbit Anti-Igfbp2 | Beyotime | Cat. AF7191 | WB,1:500 |
| Mouse Anti-Irs1 | Santa Cruz | Cat. sc-559 | WB,1:1000 |
| Rabbit Anti-Ir | Cell Signaling Technology | Cat. #3025 | WB,1:500 |
| Rabbit Anti-Lepr | Zen Bio | Cat. #R381839 | WB,1:500 |
| Rabbit Anti-p-Akt (﻿Ser473) | Cell Signaling Technology | Cat. #4060 | WB,1:1000 |
| Rabbit Anti-p-Ampkα (Thr172) | Cell Signaling Technology | Cat. #50081 | WB,1:1000 |
| ﻿Rabbit Anti-p-Irβ (Tyr1150/1151) | Cell Signaling Technology | Cat. #3024 | WB,1:1000 |
| Rabbit Anti-p-Irs1 (Tyr 608) | Merck Millipore | Cat. 09-432 | WB,1:1000 |
| Rabbit Anti-Pparγ (Pparg) | Cell Signaling Technology | Cat. #2443 | WB,1:1000 |
| Rabbit Anti-RNA polymerase II CTD repeat YSPTSPS (phospho S2) antibody | Abcam | Ab5095 | CHIP, 4 µg for 10^6^ Cells |
| Mouse Anti-p16 | Santa Cruz | Cat. sc-56330 | WB,1:500 |
| Mouse Anti-p21 | Santa Cruz | Cat. sc-6246 | WB,1:500 |
| Mouse Anti-p27 | Santa Cruz | Cat. sc-1641 | WB,1:500 |
| Mouse Anti-p53 | Santa Cruz | Cat. sc-126 | WB,1:500 |
| Mouse Anti-Srebp1 (Srebf1) | Santa Cruz | Cat. sc-365513 | WB,1:500 |
| Rabbit Anti-β-actin | Cell Signaling Technology | Cat. #4970 | WB,1:1000 |
